# A survey of Transformer applications for histopathological image analysis: New developments and future directions

**DOI:** 10.1186/s12938-023-01157-0

**Published:** 2023-09-25

**Authors:** Chukwuemeka Clinton Atabansi, Jing Nie, Haijun Liu, Qianqian Song, Lingfeng Yan, Xichuan Zhou

**Affiliations:** School of Microelectronics and Communication Engineering, Chongqing University, Chongqing, 400044 China

**Keywords:** Transformer, Histopathological imaging, CNN, Whole slide image, Survival analysis, Digital pathology

## Abstract

Transformers have been widely used in many computer vision challenges and have shown the capability of producing better results than convolutional neural networks (CNNs). Taking advantage of capturing long-range contextual information and learning more complex relations in the image data, Transformers have been used and applied to histopathological image processing tasks. In this survey, we make an effort to present a thorough analysis of the uses of Transformers in histopathological image analysis, covering several topics, from the newly built Transformer models to unresolved challenges. To be more precise, we first begin by outlining the fundamental principles of the attention mechanism included in Transformer models and other key frameworks. Second, we analyze Transformer-based applications in the histopathological imaging domain and provide a thorough evaluation of more than 100 research publications across different downstream tasks to cover the most recent innovations, including survival analysis and prediction, segmentation, classification, detection, and representation. Within this survey work, we also compare the performance of CNN-based techniques to Transformers based on recently published papers, highlight major challenges, and provide interesting future research directions. Despite the outstanding performance of the Transformer-based architectures in a number of papers reviewed in this survey, we anticipate that further improvements and exploration of Transformers in the histopathological imaging domain are still required in the future. We hope that this survey paper will give readers in this field of study a thorough understanding of Transformer-based techniques in histopathological image analysis, and an up-to-date paper list summary will be provided at https://github.com/S-domain/Survey-Paper.

## Introduction

**Fig. 1:**
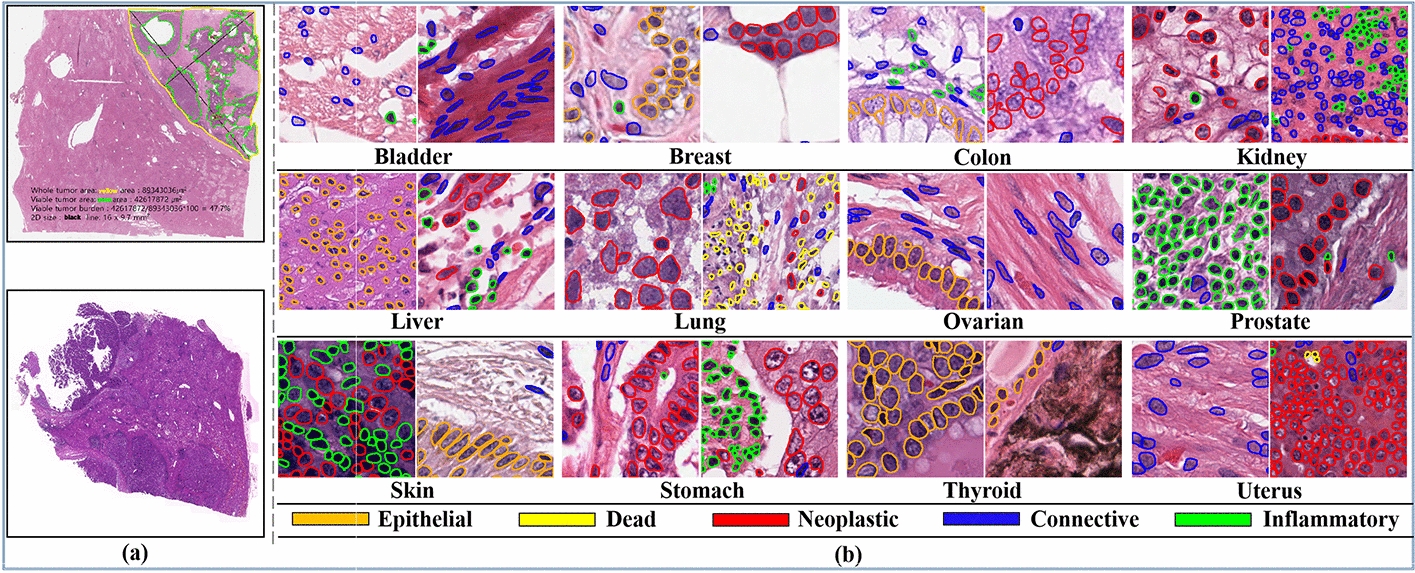
Some samples of histopathological images. **a** Whole slide images (WSIs). **b** Annotated PanNuke dataset from different tissue types for nuclei instance classification and segmentation

Histopathological imaging has been regarded as a technique for identifying nearly all types of cancers since it provides a more thorough understanding of the diseases [1, 2]. They are a very important source of primary information in clinical domains, which assists pathologists in performing cancer diagnosis. Histopathological images are mostly used for cancer grading and offer more detailed information for diagnosis when compared to other medical imaging techniques, including magnetic resonance imaging (MRI), computerized tomography (CT), transrectal ultrasound (TRUS), mammography, and many others, and diseases are also examined by identifying the cells and tissue present in lesions [1, 3]. For various cancer types, pathologists may choose treatment plans based on histopathological images coupled with genomic records. With the recent development and deployment of digital slide scanners in different clinical areas, the digitization of histopathological slides (i.e., whole slide images (WSIs)) into gigapixel images is becoming more prevalent. In computational pathology, histopathological slides (WSIs) display a hierarchical formation of visual tokens across different resolutions and can have a pixel size up to 160,000 × 160,000 pixels at 20×magnification. Figure 1 shows some samples of histopathological slides and some annotated patches extracted from the slides that contain different tissue types.

**Fig. 2:**
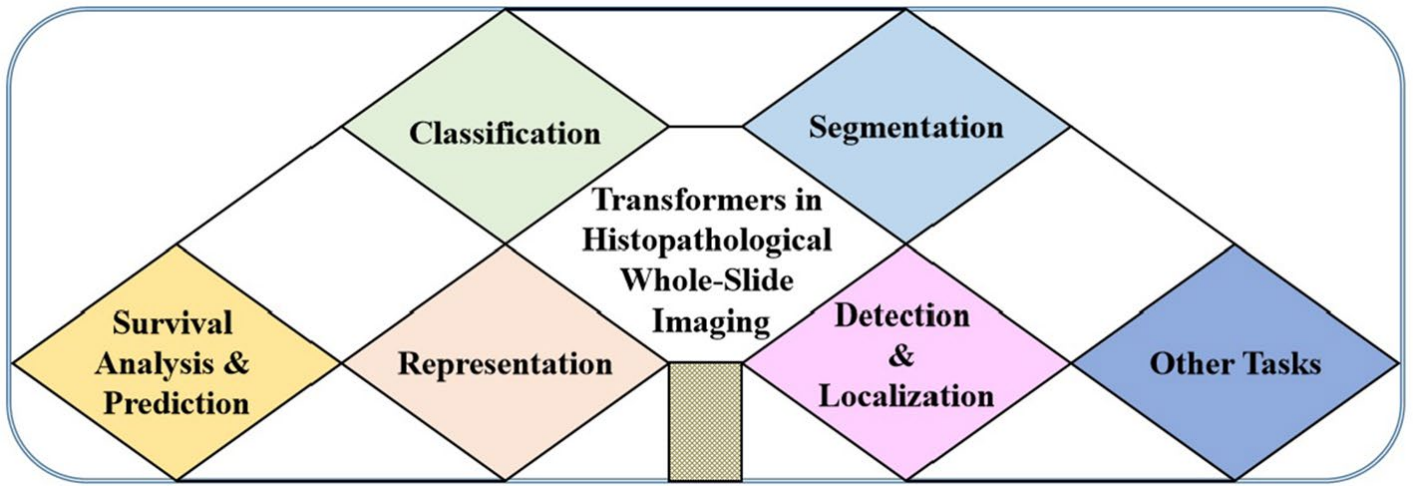
Current transformer applications in histopathological image analysis, as surveyed in this research work

The technique of digitizing histopathological images, known as digital pathology, creates a new approach to collecting image data for artificial intelligence technologies. In recent years, artificial intelligence techniques that process and analyze histopathological images have become more common in both scientific research and clinical settings. This is primarily due to the rise of deep learning, especially convolutional neural networks (CNNs), which have achieved outstanding results in many computer vision tasks [4–6]. Recently, an alternative CAD system that is capable of modeling long-range pixel information, such as transformers, has been developed. Transformers [7] have emerged as one of the most recent technological developments in deep learning for achieving robust results in many computer vision tasks. It was first built as a robust example of using deep learning techniques to tackle sequential inference tasks in natural language processing (NLP). Dosovitskiy [8] et al. introduced a vision transformer (ViT)-based architecture for image classification tasks, demonstrating that relying on CNNs for image classifications is unnecessary and that a pure transformer applied straight to sequences of image patches can get excellent results. Other than images and NLP tasks, transformers have also been adopted and applied to other deep learning domains, including autonomous driving [9], video classification [10], security [11], general audio representations [12], audio–video synchronization [13], mobile devices [14] and so on. Motivated by this innovation, several studies have adopted a variety of approaches to solve different deep learning challenges, including CNN- and transformer-based approaches, but it still remains unclear whether ViT architectures can produce better results than CNNs for histopathological image analysis. Transformers, like any other deep learning or machine learning technique, have pros and cons. Besides, transformers, unlike CNN-based approaches, are devoid of convolution-induced biases, which enables them to capture long-range contextual information and learn more complex relations in the image data. This is advantageous in histopathological imaging, where it is critical to consider not just the region of interest, but also the neighboring tissues when diagnosing a particular disease. Transformers, on the other hand, are data-demanding and require greater computing effort. This can be a difficult problem, especially in the field of histopathological imaging, where resources may be inadequate due to concerns about patient privacy. At present, many studies have been conducted in the field of histopathological imaging using transformer-based approaches, including image segmentation [2, 15], classification [16, 17], detection [18, 19], representation [20–22], cross-modal retrieval [23], image generation [24], survival analysis [25] and survival prediction [26]. Figure 2 displays current transformer applications in histopathological image analysis, as surveyed in this research work, which will be further explored in ''Current progress'' Sect.

However, based on the recently published studies, it has been shown that transformer architectures have the capacity to achieve higher performance on various histopathological imaging tasks than the previous models. 

**Fig. 3:**
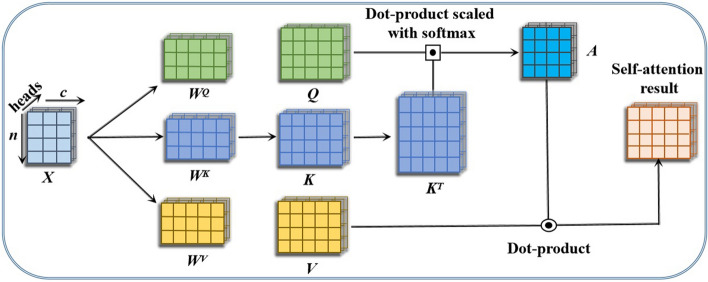
A schematic demonstration of the self-attention mechanism

Moreover, the primary aim of the paper is to provide a thorough review of transformer applications in the histopathological imaging field and demonstrate how transformers are applied to a variety of tasks. In particular, it provides readers in this field of study with a thorough understanding of transformer-based techniques in histopathological image analysis and also establishes the foundation for future innovation to improve the performance of transformer architectures in this domain. To this end, our key contributions include: (1) this work provides a thorough evaluation of more than 100 research publications across the histopathological imaging field to cover the most recent innovations; (2) it provides a thorough overview of the entire domain by classifying the research papers according to how they apply to histopathological imaging, as shown in Fig. 2; (3) it classified each of these applications, pointed out task-specific challenges, and highlighted the approaches used to address them based on the proposed work, as demonstrated in Tables 1, 2, 3, 4, and 5 in ''Current progress'' , ''Discussion'' Section it provides a thorough analysis of designing transformer-based approaches for handling more difficult real-world challenges and also compares transformers with CNN-based models based on recently published works. The remainder of this survey paper is structured as follows: ''Background'' Sect. Provides a brief background on the study and basic components of transformers. In Current progressSect. Current applications of transformers in histopathological image analysis are investigated. The discussions and conclusion are covered in Discussion, ''Conclusion ''Sect. Respectively.

## Background

Over the years, histopathological imaging computer-aided diagnosis (CAD) systems have witnessed a lot of technological advancement following the advent of transformer architectures. However, in this part, we will give a quick overview of CNN-based approaches and outline the basic operating principles together with their main advantages and drawbacks in the field of histopathological imaging. In addition, we will also discuss the fundamental ideas that underlie the success of the transformer-based techniques and then provide further information in subsequent sections. Finally, we compare the CNN methods versus the transformer methods.

### CNN applications in histopathological image analysis

For some years now, CNNs have proven to be good at analyzing image data and are the most widely used deep learning networks for many medical and clinical challenges, especially histopathological imaging. This is as a result of the strong prior that the convolution operations impose on the weights, forcing the identical weights to be shared across each and every pixel [27]. The major advantage of CNN-based approaches compared to previous architectures is their ability to automatically identify important features in an image without any form of human oversight. The process of building any CNN architecture for histopathological image analysis is a collaborative effort between researchers and medical professionals. These innovations are primarily driven by a lot of architectural advancements, improved loss functions, the accessibility of specialized hardware devices, and publicly accessible libraries created for specific purposes. Therefore, we direct readers who are interested in this research direction to some previously published survey papers on CNN applications in the histopathological imaging field [4–6]. Although CNN-based techniques have experienced a lot of architectural improvements over the years, their ability to be applied to the full range of histopathological image tasks is also constrained by their dependency on huge amounts of labeled datasets. The study of histopathological imaging for different clinical tasks has also been cross-pollinated by the CNN models [28–30], and they sometimes function as black box solutions and are typically more difficult to explain. However, the success of CNN-based methods is primarily due to their capacity to extract useful information from input images, doing away with the necessity for conventional manual image processing techniques. Despite increasing the receptive field, they still face a lot of challenges in modeling long-range information as well as spatial dependencies due to their weight sharing and inductive bias locality. The local nature of the convolutional operations in CNNs is the major challenge associated with CNN-based techniques, as it prevents them from capturing long-range semantic dependencies from the given input images. Thus, an alternative CAD system that is capable of modeling long-range pixel information, such as transformers, is required to achieve more robust results than the previous models.

### Transformers

#### Basics

Transformer-based architectures are the most advanced technique for handling sequences. They make use of attention mechanisms due to their capacity to model long-range semantic information. Besides, they also make use of an encoder–decoder design strategy that produces an output without relying on recurrence and convolutions. As a result, we first begin by giving a brief introduction to the basic ideas behind the attention mechanism, followed by a comprehensive explanation of how the transformer operates.

#### Attention mechanism

The attention mechanism evolved naturally from sequence-related challenges. Nowadays, it is often used to extract unimportant information from the data while concentrating on the relevant portions of the data, and it can be used for a number of deep learning architectures across different clinical domains and downstream tasks. An attention mechanism was initially developed to boost machine translation encoder–decoder performance. It was initially introduced by Bahdanau et al. [31] for the language translation task to tackle the bottleneck that results from the use of a fixed-length encoding vector, where the decoder would have minimal access to the information delivered by the input. This is viewed as being especially troublesome for long or sophisticated sequences because the representation’s dimensionality would be limited to match that of unsophisticated or shorter sequences. 

(i) Attention mechanisms in computer vision tasks:

The concept of emulating human attention emerged in the computer vision domain in an attempt to minimize the computational problem of image processing while increasing accuracy by adding a model that only focused on certain portions of images rather than the whole image. However, the attention mechanisms we employ today in our various models originated in the field of NLP. Several studies have been proposed in the past to incorporate attention mechanisms into their architecture. For example, the work in [32] instead focuses on the interaction between channels and develops a new attention mechanism framework known as squeeze-and-excitation that explicitly models the interdependencies between channels and adaptively recalibrates channel-wise feature responses. In contrast to Bahdanau attention, the attention mechanism, as proposed in  [7], has been reconstructed as a function that uses values, keys, and queries that are attained from the module’s input vectors. In practice, the values and keys are constructed together into matrices V and K, while the attention function is computed simultaneously on a set of queries and arranged together into a matrix Q. Then, the output function is determined as a weighted sum of values, where each value of the weight is computed as the attention between queries and keys, respectively. In addition, the operation of self-attention, as illustrated in Fig. 3, is typically performed in matrix formation in order to speed up the parallel calculation. Additionally, in order to quickly demonstrate a clear picture of the self-attention mechanism, we begin by defining it in an element-wise manner. Let ${x_i}\in \mathbb {R}^c,i=1,..,m,$ be the input image, and the corresponding vectors generated by the parameters (i.e., $W^q, W^k, \,and \, W^v$ ) be query ${q_i}\in \mathbb {R}^g_q$, key ${k_i}\in \mathbb {R}^g_k$, and value ${v_i}\in \mathbb {R}^g_v$, respectively. Again, $g_q$, $g_k$, and $g_v$ represent the number of features learned from $x_i$ and also the sizes of $q_i$, $k_i$, and $v_i$, respectively.

1 $$\begin{aligned} \left\{ \begin{array}{llll} q_i = x_i \times W^q, where \, {W^q}\in \mathbb {R}^{c \times g_q}, \\ k_i = x_i \times W^k, where \, {W^k}\in \mathbb {R}^{c \times g_k}, \\ v_i = x_i \times W^v, where \, {W^v}\in \mathbb {R}^{c \times g_v}, \\ \,\,\,\,\,\,g_q = g_k. \end{array} \right. \end{aligned}$$

A softmax function is used to calculate the weights $\beta _{ij}$ and is represented by the following equation:

2 $$\begin{aligned} \beta _{ij}~= ~& {} \Gamma \left( \frac{\beta ^\prime _{ij}}{\sqrt{g_k}}\right) = \frac{exp\left( \frac{\beta ^\prime _{ij}}{\sqrt{g_k}}\right) }{\sum _j exp\left( \frac{\beta ^\prime _{ij}}{\sqrt{g_k}}\right) }. \end{aligned}$$

3 $$\begin{aligned} \beta ^\prime _{ij}=~ & {} q_i \times k^T_j, \end{aligned}$$

where $\Gamma$ represents the softmax function and $\beta ^\prime _{ij}$ computes the contribution of the $j{th}$ input element to the $i{th}$ output element. Throughout this process, $\beta ^\prime _{ij}$ is considered to be the attention attributed to the factor $v_i$. As a result, the final resultant attention can be calculated as a weighted total of each and every value, as shown below:

4 $$\begin{aligned} y_i = \sum_j {\beta_{ij}} \times v_j. \end{aligned}$$

In addition, it is reasonable to extend element-wise self-attention into matrices. However, for each input $x_i$, parallel matrix computation is commonly used to produce and create the query $q_i$, key $k_i$, and value $v_i$, respectively. Matrices can be formed by stacking up the input $x_i$, value $v_i$, query $q_i$, and key $k_i$, accordingly. Let ${X}\in \mathbb {R}^{n \times c}$ be the input matrix, and the value, query, and key matrices be V, Q, and K, respectively. The number of samples is represented by n, and each individual matrix is made up of the components (i.e., $X = [x_1; x_2; x_3; \cdots ;x_n]^T)$. Therefore, the attention matrix *A* and resultant matrix *Y* are now computed as shown below:

5 $$\begin{aligned} A ~=~ \, & {} \Gamma \left( \frac{Q \times K^T}{\sqrt{g_k}}\right) \in \mathbb {R}^{n \times n}, \end{aligned}$$

6 $$\begin{aligned} Y=~\, & {} A \times V \in \mathbb {R}^{n \times g_v}, \end{aligned}$$

The authors [7] created a different form of attention mechanism known as multi-head self-attention (MHSA). They demonstrated that applying several self-attentions to the same input allows for a more efficient acquisition of hierarchical information. However, in the mechanism, $$h~(i.e.,~h = 8)$$ distinct attention heads were generated, each with a unique set of weight matrices (*W*(*Q*), *W*(*K*),  and *W*(*V*)). The key, value, and query matrices are then created for each attention head by multiplying the input matrix by each of the weight matrices $$(W^Q, W^K,$$ and $$W^V)$$. Again, these query, key, and value matrices are subjected to attention mechanisms in order to produce an output matrix from each attention head. In addition, the output of the MHSA layer is produced by concatenating the output matrix acquired from each attention head (*h*) and the dot product with the weight $$(W^O)$$. Finally, given self-attentions (heads) denoted as *h*, the system produces the desired output result by integrating the computed attentions as illustrated in the equation below:

7 $$\begin{aligned} Y_i~=~\, & {} A(Q \times W^Q_i, K \times W^K_i, V \times W^V_i), \end{aligned}$$

8 $$\begin{aligned} M_H(Q,K,V)~=~ & {} \,f_{C}(Y_1, Y_2, Y_3, \cdots , Y_h)W{^O}, \end{aligned}$$

where $M_H$ denotes the multi-head self-attention operator and $f_{C}$ is the concatenating function. The linear projection matrices $W^Q_i, W^K_i$, and $W^V_i$ map the *Q*, *K*, and *V* matrices into the appropriate subspaces.

#### Transformer architecture

**Fig. 4:**
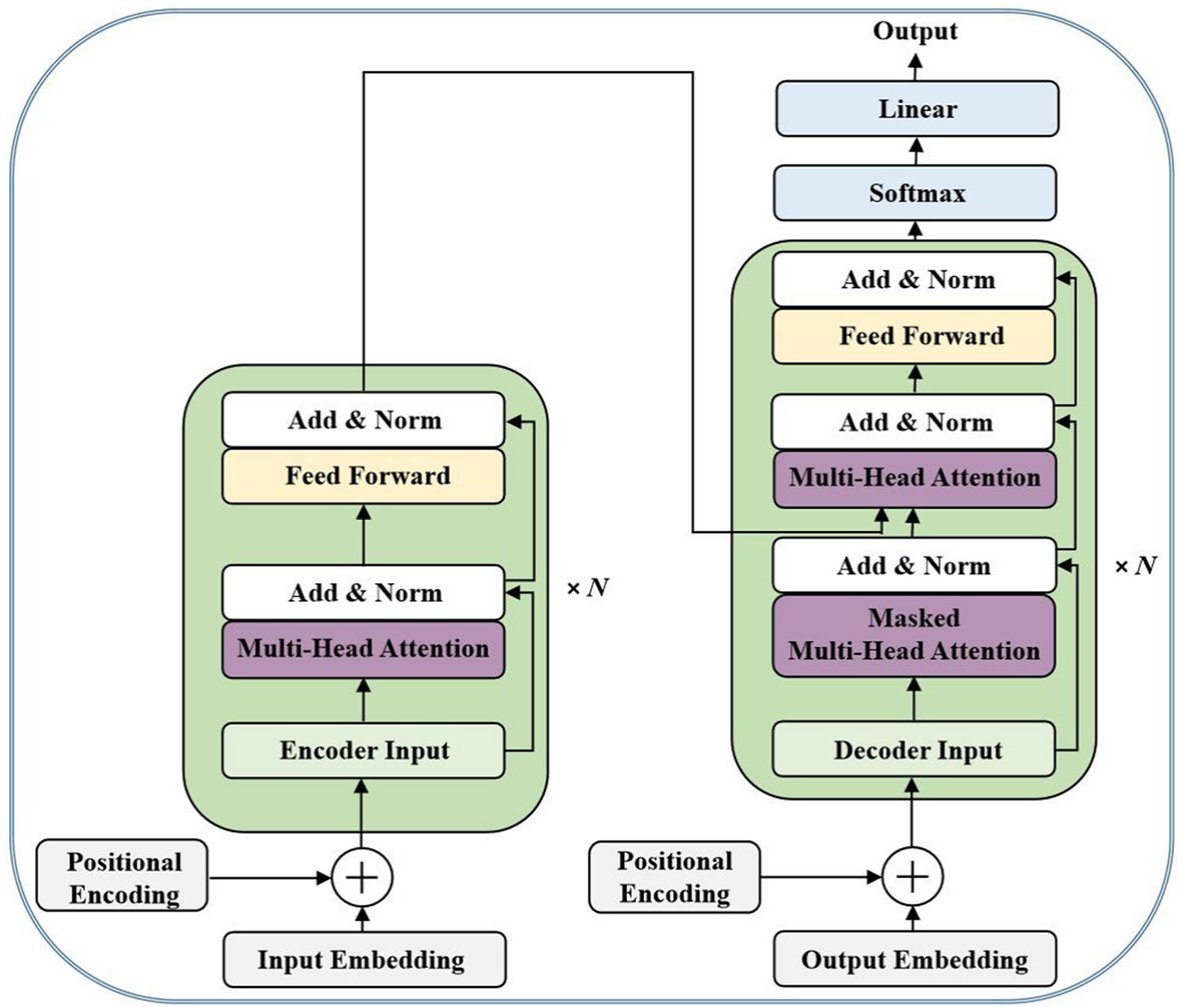
A schematic demonstration of a standard transformer architecture

Transformers are generally designed to handle sequence-related tasks while also dealing with long-term dependencies. In the paper titled “Attention Is All You Need” [7], the authors introduced a standard transformer architecture that employs an encoder–decoder formation, as shown in Fig. 4, which will be discussed further in the subsequent sections. In the architecture, the encoder framework converts an input sequence $(x_1, x_2, x_3,...,x_n )$ into a series of continuous representations (i.e., an output sequence) z = $(z_1, z_2, z_3,...,z_n)$. The decoder then produces the resultant sequence $(y_1, y_2, y_3,...,y_m )$ one component at a time from the encoded representation z, using the previous output as additional input when generating the next. The transformer follows this general architectural framework, which employs different layers in both the encoder and decoder modules, as demonstrated on the left and right sides of Fig. 4.

(i) Transformer encoder

Transformer architectures, as shown in Fig. 4 mainly consist of both encoder and decoder blocks. The encoder is composed of $N = 6$ identical layers built on top of one another that extract features from the input sequence. Each layer is made up of two sub-layers known as the feed-forward network layer (FFNL) and the multi-head self-attention mechanism (MHSA). Again, residual connections were employed across each of the sub-layers, followed by layer normalization. First, the multi-head attention is computed in each block, followed by a layer-wise normalization block. The sum of the multi-head attention input and output is computed primarily using layer-wise normalization. After applying a feed-forward layer, the input and output of the feed-forward layer are summed together using layer-wise normalization. 

(ii) Transformer decoder

The transformer decoder shown on the right-hand side of Fig. 4 uses the extracted features to generate the output sequence. It consists of $N = 6$ identical layers with a few modifications. An additional sub-layer block is added on top of the encoded output, which carries out multi-head attention over the encoder stack output. Since the prediction is based on a known state, masking was utilized in the first self-attention block to prevent further contributions to the state of the preceding position. In addition, after the decoder’s output layer, a linear and a softmax layer are added to produce the final result.

#### Vision transformer (ViT)

**Fig. 5:**
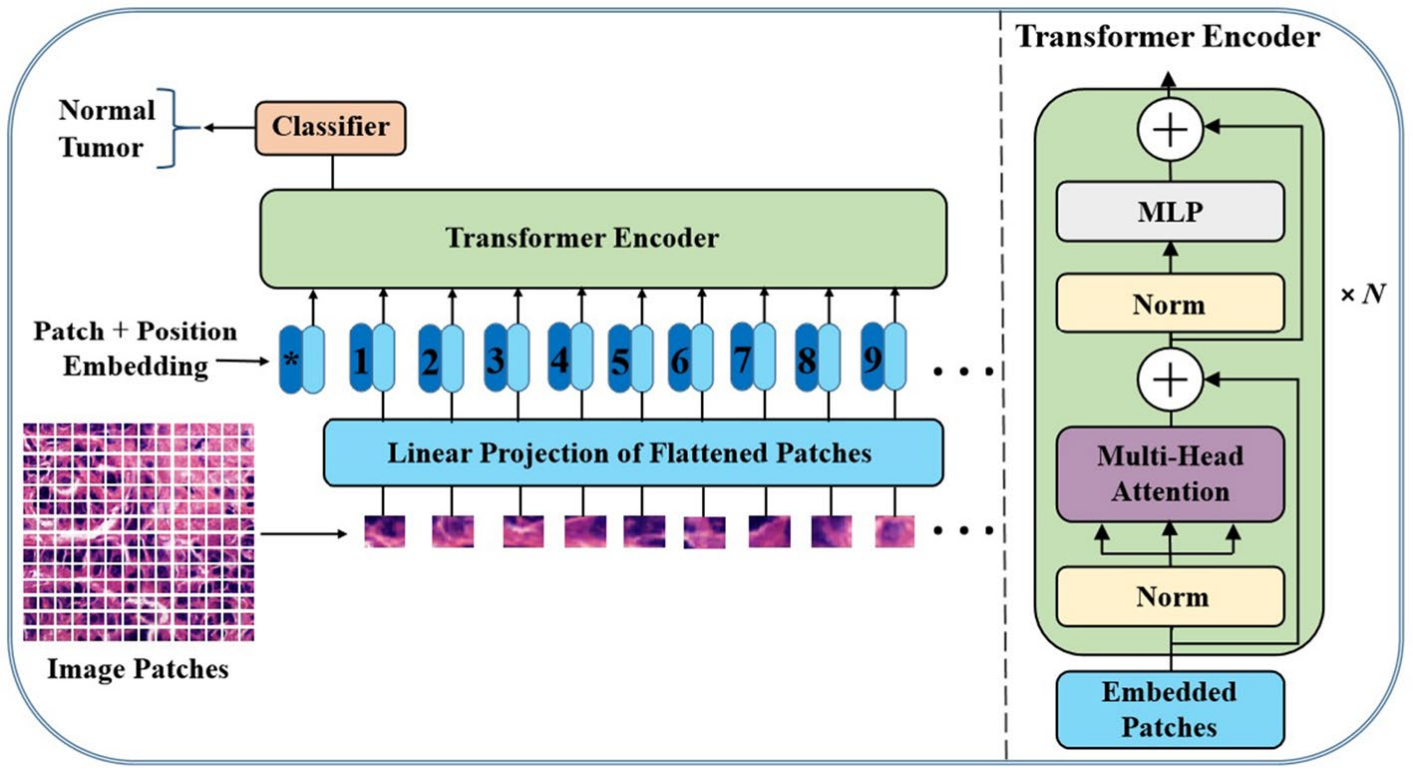
A schematic diagram of a standard ViT model. Sequential image patches are used as the input, which is then processed with a transformer encoder and uses an MLP head module to generate a class prediction

Transformers were initially introduced in NLP tasks where the objective was to understand the text and draw relevant and useful conclusions. Transformer architectures have accomplished significant results and have become a de facto standard in the field of NLP because of their generalization abilities and simplicity. Following their success in NLP tasks, researchers in this domain have made numerous attempts to adapt transformer architectures to various vision challenges. Among the most common transformer-based architectures in vision that have been established are the DETR [33], Swin-transformer [34], ViT [8], DeiT [35], and BEiT [36]. In [33], the authors were the first to make use of transformers in computer vision for object detection tasks. The proposed architecture, known as DETR, focuses on a transformer encoder–decoder architecture and a set-based global loss that forces unique predictions through bipartite matching. Unlike other traditional object detection approaches that rely heavily on handcrafted techniques, the DEtection transformer does not need any special layers, which makes it easy to replicate in any model that has common transformer and CNN classes. On the other hand, it is simple to generalize and create unified panoptic segmentation. In 2021, Dosovitskiy et al. [8] introduced a vision-based transformer known colloquially as ViT, stating that CNNs were no longer required and that a pure transformer architecture applied instantly to sequences of image patches can produce robust results, particularly on image classification problems. The input image, as presented in Fig. 5, is split into a number of patches, each of which is encoded spatially to provide spatial information using a positional encoding technique. The ViTs have produced better or even higher results, outperforming state-of-the art (SOTA) CNNs for many downstream tasks, especially when pre-trained on huge datasets. To this end, transformer architectures require more training data to obtain comparable results or even higher than CNNs, and more details will be provided in subsequent sections.

#### Pros and cons of a transformer architecture

Transformers have been widely used in many computer vision challenges and have shown the capability of producing better results than other deep learning techniques. Some of the advantages of transformers in computer vision tasks include efficient parallel processing, adaptability with variable-length sequences, effective handling of global dependencies, higher network capacity, and so on. Due to the attention mechanisms incorporated into the networks, they can process sequences in parallel and also handle global dependencies, making them more efficient and faster than standard sequential networks such as recurrent neural networks. In addition, transformer architectures also produce robust results on NLP tasks due to higher network capacity and the ability to capture complicated relationships in sequential data. Despite the fact that transformers can enable higher network capacity and learn more complex relations in the image data, they also have some drawbacks. Some of the disadvantages of transformers in computer vision tasks include high computing costs, overfitting vulnerability, data inefficiencies, and so on. Transformers are more resource-intensive than any other deep learning technique due to the self-attention mechanism built into the networks, which necessitates a lot of computation as well as training time. Furthermore, insufficient data to train the model effectively is another notable disadvantage of transformers, which can pose a lot of problems in NLP tasks where there is a limited amount of labeled data.

### Transformer methods versus CNN methods

Over the years, CNNs have shown outstanding performances for histopathological image analysis, while transformers such as ViTs have produced better or even higher results, outperforming SOTA CNNs for many downstream tasks, especially when pre-trained on huge datasets. CNN architectures are more mature and make use of pixel arrays, so they are easier to implement, study, and train when compared to transformer architectures. During training, as the depth of the networks increases, the receptive field of CNNs significantly widens; therefore, the features mined at lower stages differ significantly from those at later stages. Besides, CNNs make use of convolution, a “local” technique limited to a tiny area of an image, which makes them more advantageous in capturing local semantic structures. The feature maps created by the CNNs through the convolution process using these trainable convolutional filters, which are hidden representations of the true image, only affect a tiny portion of the image at a time. Additionally, CNNs are also limited in capturing long-distance correlations between image regions due to their small receptive field. On the other hand, transformer architectures make use of a self-attention mechanism, a “global” technique since it gathers relevant information from the entire image. This enables them to effectively capture more distant and important information in an image. The representation in transformer architectures is similar in every layer and can gather global information early owing to self-attention. Again, the MHSA in particular provides a global receptive field, which results in identical representations in distinct numbers of layers. Moreover, all attention outputs are linearly concatenated to the appropriate dimensions by the MHSA layer, and the block of each layer of the MHSA has the capacity of aggregating features globally to produce accurate knowledge of long-distance interactions. To this end, transformer architectures require more training data to obtain comparable results or even higher than CNNs, and more details will be provided in subsequent sections.

## Current progress

Vision transformers (ViTs) have been generally used for a variety of clinical purposes. However, in this section, we will first discuss the searching procedures used to obtain all the papers reviewed in this survey (see ''Article searching and selection procedures'' Sect.). Then, we will present and discuss different ways of employing transformers for histopathological imaging in ''Different ways of employing Transformers for histopathological imaging'' Sect. Finally, the current transformer applications in histopathological image analysis, as shown in Fig. 2, are discussed in '' Current transformer applications in histopathological imaging'' Sect.

### Article searching and selection procedures

**Fig. 6:**
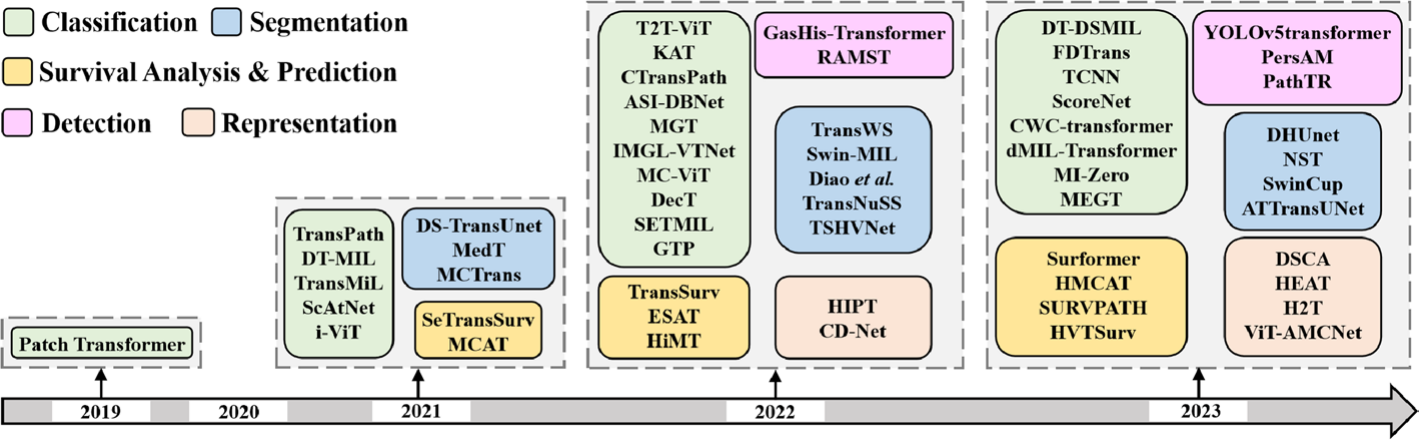
Transformer-based architectures for histopathological image analysis. The figure shows some of the existing approaches for different downstream tasks, including segmentation, survival analysis and prediction, representation, detection, and classification

This section presents a brief discussion of the methods used in searching for and selecting the research papers. The newly built architectures, as shown in Fig. 6, are classified based on their learning tasks.

**Fig. 7:**
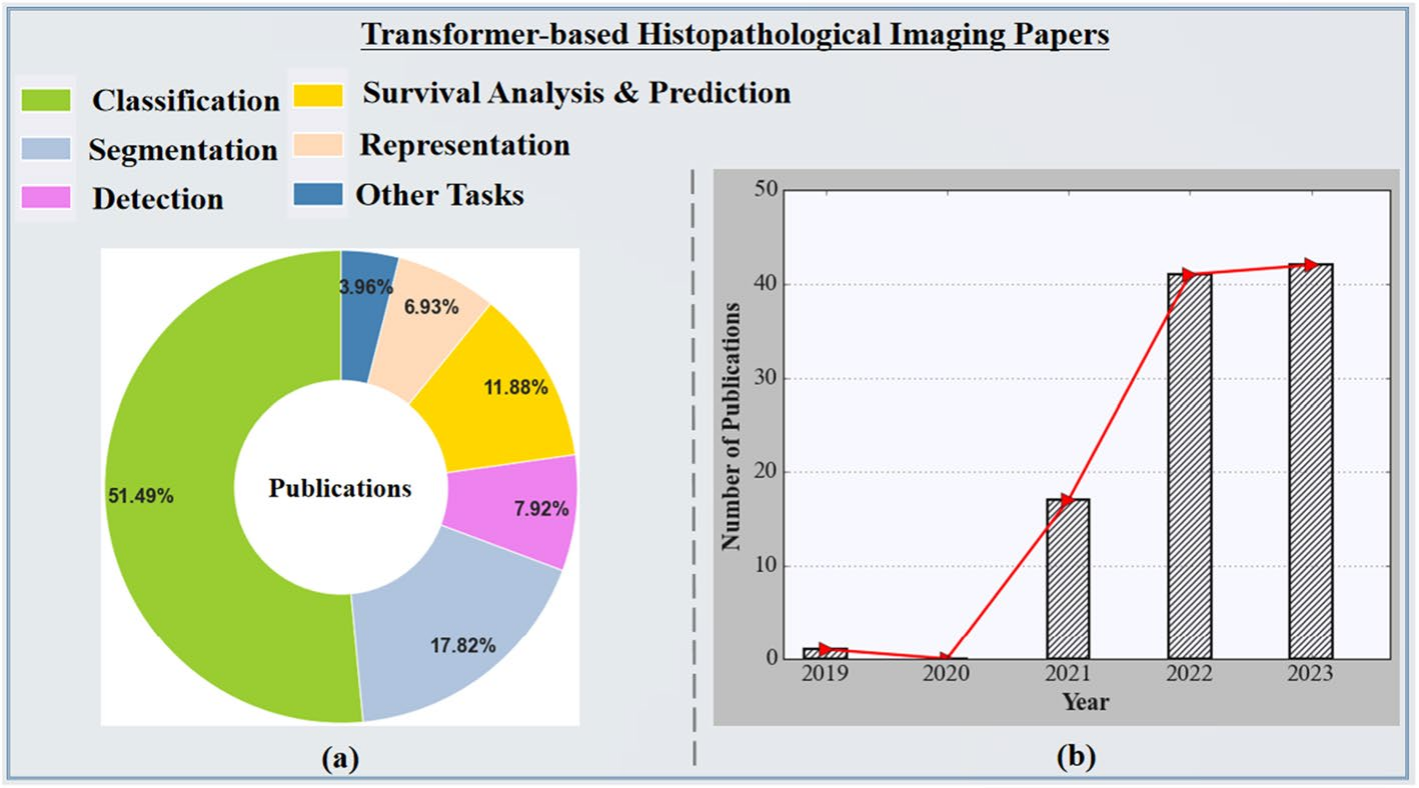
The chart **a** displays the statistics of the papers presented in this survey according to histopathological imaging problem settings. The rightmost figure **b** demonstrates consistent growth in recent development (from 2019 to July 2023)

As demonstrated in Fig. 7(b), the histopathological imaging domain has been slightly impacted by transformer-based architectures since the inception of the first ViT architecture. Figure 7(a) displays the statistics of the papers presented in this survey according to histopathological imaging problem settings. In particular, we explore publications from Science Direct, Springer, Xplore, PubMed, IEEE, and conference proceeding papers, especially those from conferences on medical imaging like SPIE, RSNA, IPMI, MICCAI, ISBI, and so on. In addition, we use Google Scholar to search for paper references and manuscripts. As a result of our search queries using various keywords such as vision transformers, transformers in medical imaging, transformers in histopathological imaging, transformers in image classification and segmentation, and so on, we found more than a thousand papers about the transformer, some of which are from the fields of natural imaging or language studies. Again, we construct the concepts of our survey from the self-attention and the ViT published papers, which are major milestones for the investigation of transformers in histopathological image analysis. Finally, we limited the survey research to exclusively cover transformer applications in the histopathological imaging domain. As presented in Fig. 6, we show the categorization of some recently developed models based on the learning tasks in the histopathological imaging field. Then, in Fig. 7, we show the percentage of the papers presented in this survey according to histopathological imaging problem settings and consistent growth in recent development, which will be further discussed in the following subsections.

### Different ways of employing transformers for histopathological imaging

Recently, numerous studies have been conducted on how to apply transformers for histopathological image analysis. Some studies attempted to use only pure transformers (i.e., transformers without convolution blocks (see Fig. 5), while others tried to integrate the benefits of transformers (e.g., DETR [33], ViT [8], DeiT [35], BEiT [36], Swin-transformer [34], and so on) and CNNs (e.g., EfficientNet [37], Unet [38], ResNet [39], and so on) for different downstream tasks. However, in this section, we will classify them into three distinct types, which will be further discussed in the following subsections.

(i). Pure transformers: Pure transformers, as shown in Fig. 5, are described as those ViT-based architectures that resemble the ones originally proposed by Dosovitskiy et al. [8] which typically do not include major structural adjustments. They outperform conventional CNN models in terms of scalability and efficiency at both small and large computational sizes. TransWS [40], MCAT [26], HIPT [22], PyT2T-ViT [41], and ViT-WSI [17] are some examples of pure transformer models developed for different histopathological imaging tasks.

(ii). Graph-based transformer methods: These are the types of transformer networks that introduce graphs into traditional vision transformers (see Fig. 9(a) GTP). Moreover, graphs are a common type of data structure, and there are several areas of application in which datasets can be characterized as graphs, such as biological networks, social networks, and several other types of multimedia domain-specific data. However, using graph-based learning methods is a normal practice in both histopathological and other medical image analysis. As a result, analyzing graph data can reveal important information about node classification, and the basic idea behind graph learning is to use the data graph to learn a dense representation of each and every sample, such as embeddings, while maintaining the intrinsic inter-sample relationships. Transformer, as an attention-based model, is capable of processing graph data, including aggregating node information and determining the relationship between the nodes. Dwivedi et al. [42] developed a graph transformer network (GTN) that supports the use of specific domain information as edge features and provides interpretability via self-attention modules that locate the key regions of the graphs for prediction. AMIGO [43], LA-MIL [44], Wang et al. [45], and GTP [46], MEGT [47] are some examples of graph-based transformer models that have been proposed for different histopathological image classification tasks.

**Fig. 8:**
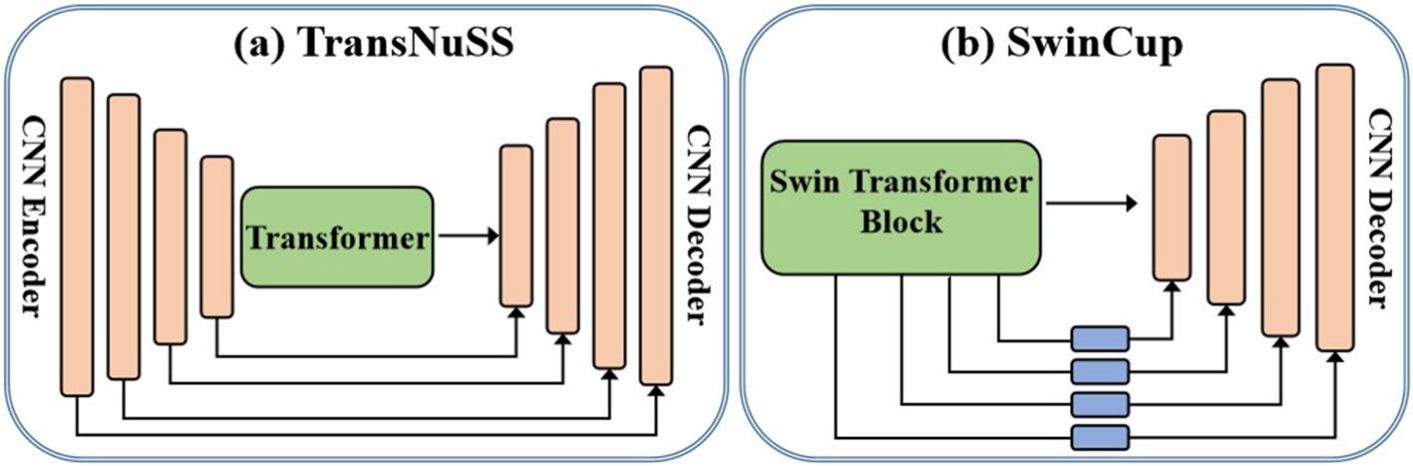
Some typical transformer U-shaped architectures

(iii). Hybrid transformer–CNN: In histopathological image analysis, there are many ways in which transformers can be combined with CNN to form a hybrid model. The simplest method is to use both in an effort to capitalize on both of their advantages. These hybrid networks either use transformer to replace some parts of the network or incorporate transformer into the entire network by using CNN as the backbone of the network. However, we find out that current research in histopathological image segmentation focuses mainly on the following three issues to develop transformers combined with the widely used U-shaped framework: firstly, transformer blocks are inserted at various positions in the U-shaped structure, as shown in Fig. 8. Secondly, employing several techniques to combine CNN and transformer networks. Finally, making use of attention mechanisms or employing multi-scale features. SeTranSurv [25], ATTransUNet [2], TCNN [1], SwinCup [48], and DHUnet [49], TransNuSS  [50] are some examples of hybrid transformer–CNN models that have been developed for different histopathological imaging tasks.

### Current transformer applications in histopathological imaging

This section presents the current applications of transformers in histopathological image analysis, such as classification, segmentation, survival analysis and prediction, representation, detection and localization, and other tasks. These applications, as demonstrated in Fig. 2, are classified based on their learning tasks.

#### Histopathological image classification

**Fig. 9:**
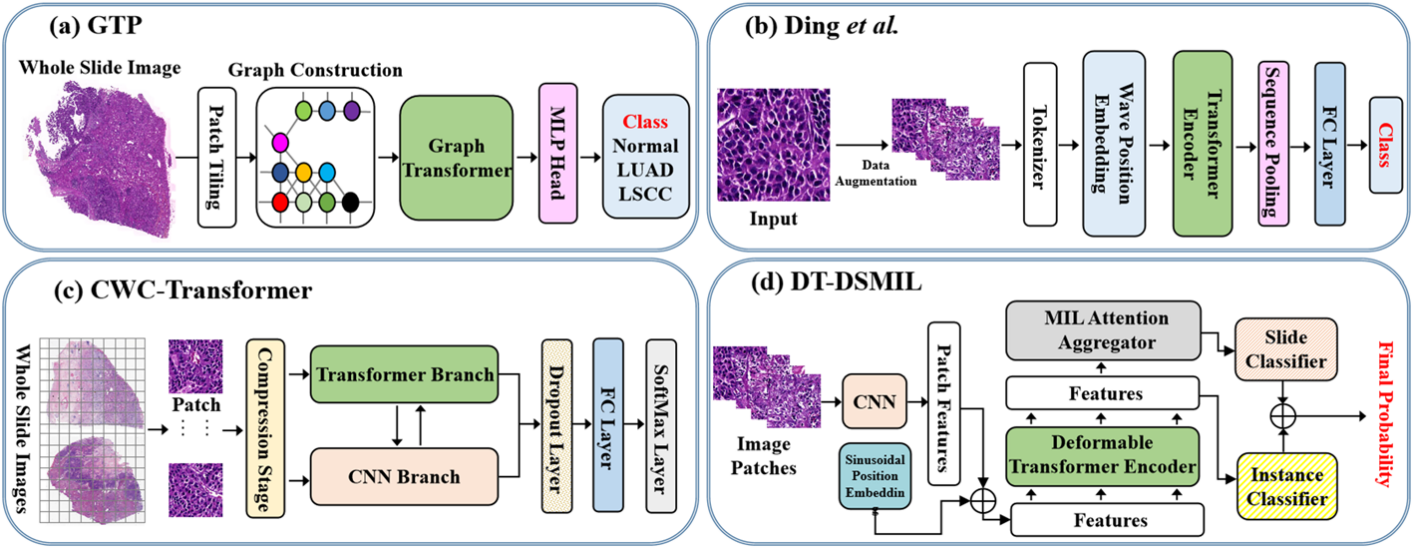
Some examples of SOTA transformer architectures for histopathological image classification

Vision transformer [8] has demonstrated remarkable performance in several natural image classification tasks since its inception. From previous and past studies, transformer-based techniques for cancer investigation and prediction are often referred to as classification tasks and can be classified into three distinct classes. Firstly, the direct application of transformer architectures to histopathological images. Secondly, making use of transformer architectures in conjunction with convolutions to learn more representative local features. Finally, making use of transformer architectures in conjunction with graph representations will help better manage data with complex sizes. This section, as demonstrated in Table. 1, will provide a thorough overview of current transformer applications for histopathological image classification. Figure 9 shows some examples of SOTA transformer architectures developed for histopathological image classification. For breast cancer histopathological image classification, DCET-Net [72] proposed a dual-stream convolution-expanded transformer architecture; Breast-Net [51] explores the ability of ensemble learning techniques using four Swin transformer architectures; HATNet [52] uses end-to-end vision transformers with a self-attention mechanism; ScoreNet [16] developed an efficient transformer-based architecture that integrates a coarse-grained global attention framework with a fine-grained local attention mechanism framework; LGVIT [73] built a local–global ViT model by introducing a new local–global MHSA mechanism and a ghost geed-forward network block into the network; dMIL-transformer [53] developed a two-stage double max–min multiple-instance learning (MIL) transformer architecture that combines both the spatial and morphological information of the cancer regions. Other than breast cancer classification, transformers have also been applied to other histopathological image cancer classification tasks, such as bone cancer classification (NRCA-FCFL [74]), brain cancer classification (ViT-WSI [17], ASI-DBNet [54], Ding et al. [55]), colorectal cancer classification (MIST [75], DT-DSMIL [56]), gastric cancer classification (IMGL-VTNet [57]), kidney subtype classification (i-ViT [59], tRNAsformer [58]), thymoma or thymic carcinoma classification (MC-ViT [76]), lung cancer classification (GTP [46], FDTrans [60]), skin cancer classification (Wang et al. [45]), and thyroid cancer classification (Wang et al. [77], PyT2T-ViT [41], Wang et al. [78]) using different transformer-based architectures. Furthermore, other transformer models such as Transmil [65], KAT [61], ViT-based unsupervised contrastive learning architecture [79], DecT [66], StoHisNet [80], CWC-transformer [63], LA-MIL [44], SETMIL [81], Prompt-MIL [67], GLAMIL [67], MaskHIT [82], HAG-MIL [68], MEGT [47], MSPT [70], and HistPathGPT [69] have also been evaluated on more than one tissue type, such as liver, prostate, breast, brain, gastric, kidney, lung, colorectal, and so on, for histopathological image classification using different transformer approaches. As shown in Fig. 9, GTP [46] introduced a graph-based transformer architecture that combines a vision transformer for processing histopathological images and a graph-based representation of a WSI for disease grade prediction. Ding et al. [55] built an improved ViT-based architecture by introducing a wavelet position embedding framework into the network to reduce the aliasing phenomenon in histopathological features brought about by smooth discontinuous feature information and downsampling operations. CWC-transformer [63] presents a two-stage network module that successfully addresses the feature extraction and spatial information loss problems in classifying WSIs. DT-DSMIL [56] proposed a weakly supervised transformer architecture that is based on MIL to do away with the time-consuming and labor-intensive manual annotations and also to handle gigapixel images at once.

**Table 1:** Transformer applications in histopathological image classification tasks

Method	Tissue	Dataset	Challenge	Highlight	ACC / F1 / AUC (%)
ScoreNet [16]	Breast	BRACS, BACH and CAMELYON16	The huge size of WSIs and the cost of exhaustive localized annotations	Efficient transformer-based architecture local and global attention mechanism	–/ 81.10 /–
BreaST-Net [51]	Breast	BreakHis	Differentiating subtypes of benign and malignant cancers	Ensemble of Swin transformers	99.60 / 99.50 / 99.40
HATNet [52]	Breast	Custom	Diagnostic variability and misdiagnosis of breast cancer	End-to-end ViTs with self-attention mechanism	71.00 / 70.00 /–
dMIL-transformer *et al.* [53]	Breast (LNM)	CAMELYON16 and 17 and the SLN-Breast	Taking into account the morphology and spatial distribution of cancerous regions	Two-stage double max–min MIL transformer architecture	89.23 / 84.83 / 91.67
ASI-DBNet [54]	Brain	UHP	Lack of precision and accuracy in grading brain tumor	An adaptive sparse interactive ResNet ViT dual network	95.24 / 95.23 / 96.83
Ding *et al.* [55]	Brain	NCT-CRC-HE, BreaKHis and LDCH	Aliasing phenomena caused by downsampling operations and smoothing discontinuous	ViT-based network with wavelet position embedding	99.01 /–/–
DT-DSMIL [56]	Colorectal	Custom	Data annotations	Weakly supervised ViT-based MIL	93.50 / 94.37 / 97.69
IMGL-VTNet [57]	Gastric	IMGL	The problem of identifying IM glands	Multi-scale deformable transformer	–/ 94.00 /–
tRNAsformer [58]	Kidney	TCGA	Gather the information needed to learn WSI representations	Transformer-based learning to predict RNA sequence expressions	96.25 / 96.25 /–
i-ViT [59]	Kidney	TCGA-KIRP	Capturing cellular and cell-layer level patterns	Instance-based Vision Transformer network	93.01 / 93.60 /–
GTP [46]	Lung	CPTAC, TCGA and NLST	Label noise	Graph-transformer with vision transformer	91.20 /–/ 97.70
FDTrans [60]	Lung	TCGA-NSCLC	Large intra-class differences and a lack of annotated datasets	Frequency domain transformer-based architecture	92.33 / 94.64 / 93.16
Yacob *et al.* [45]	Skin	Custom	Time-consuming and inter-pathologist variability	Weakly supervised approach using graph-transformer	93.50 /–/–
KAT [61]	Stomach	Gastric-2K, Endometrial-2K	Over-smoothing and High computational complexity	Kernel attention transformer	94.9 /–/ 98.30
DT-MIL [62]	Lung and breast	CPTAC-LUAD and BREAST-LNM	The problem of learning an effective WSI representation	Deformable transformer model for MIL	–/ 96.92 / 99.06
TCNN [1]	Breast, Lung, etc.	MDD and RWD	Artifacts in WSIs	Transformer with CNN	96.90 / 97.40 / 98.50
CWC-transformer [63]	Breast and Lung	CAMELYON16, TCGA-LUNG and MSK	Loss of spatial information and problems associated with feature extraction in WSI	Combination of transformer and CNN	92.59 /–/ 94.88
TransPath [64]	Breast, Lung, etc.	TCGA, PAIP, PatchCam, etc.	Data annotation	Self-supervised learning transformer-based network	95.85 / 95.82 / 97.79
TransMIL [65]	Breast, Lung and Kidney	CAMELYON16, TCGA (NSCLC and RCC)	Correlation among different instances, Huge size and the lack of pixel-level annotations	Transformer-based multiple-instance learning (MIL)	94.66 /–/ 98.82
DecT [66]	Breast, Endometrium	BreakHis, BACH, and UC	Not taking into account the staining properties of histopathological images	Color deconvolution with transformer architecture	93.02 / 93.89 /–
LA-MIL [44]	Colorectal and stomach	TCGA-CRC and TCGA-STAD	Quadratic complexity of transformer architectures with respect to the sequence length	MIL local attention graph-based transformer model	–
Prompt-MIL [67]	Breast and colorectal	TCGA(BRCA and CRC and BRIGHT	Overfitting problems and a lack of annotated data	Prompt Tuning MIL transformer	93.47 /–/–
HAG-MIL [68]	Breast, Gastric, Lung, etc.	CAMELYON16, IMGC, TCGA-RCC and NSCLC	The difficulties in locating the most discriminative patches	Hierarchical attention-guided MIL transformer framework	91.40 / 89.40 / 98.20
MI-Zero [69]	Breast, cell, and lung	TCGA (BRCA, NSCLC and RCC), etc.	Computational issues and a scarcity of large-scale publicly available datasets	Transformer-based visual language pre-trained MI zero-shot transfer	70.20 /–/ –
HAG-MIL [69]	Breast, cell, and lung	TCGA (BRCA, NSCLC and RCC), etc.	Computational issues and a scarcity of large-scale publicly available datasets	Transformer-based visual language pre-trained MI zero-shot transfer	70.20 /–/ –
MEGT [47]	Kidney and breast	TCGA-RCC and CAMELYON16	The problem of learning multi-scale image representation from large images like gigapixel WSIs	Multi-scale efficient graph transformer-based network	96.91 / 96.26 / 97.30
MSPT [70]	Breast, and lung	TCGA-NSCLC and CAMELYON16	The problem of uneven representation between the negative and positive instances in bags	Multi-scale prototypical transformer-based network	95.36 /–/ 98.69
GLAMIL [71]	Breast, lung, and kidney	TCGA(RCC and NSCLC) and CAMELYON16	Overfitting, WSI-level feature aggregation, and imbalanced data challenges	Local-to-global spatial learning	95.01 /–/ 99.26

To this end, structural improvements, newly built transformer architectures, CNN backbones, pre-training, multiple-instance learning, and ensembling learning techniques are among the numerous innovations included in these transformer architectures for a wide range of tasks. As listed in Table 1, even though pure transformers, transformers with graphs, and hybrid transformers perform exceptionally well in a number of papers surveyed, such as breast and lung cancer classification, further improvement is still required in future research. On the whole, we therefore summarize the transformer applications for histopathological image classification as follows: firstly, transformer architectures have obtained equal or superior results in many classification tasks in comparison with CNN-based models. Secondly, transformer architectures are somewhat limited in their application, particularly in the field of histopathological imaging, because of their desire for extensive annotated datasets. However, an alternative approach to resolving this challenge could be pre-training. Thirdly, it is computationally expensive to train transformer models using gigapixel images. Therefore, in order to boost their performance, it is crucial to lower the computational cost of the model and create lightweight architectures. Fourthly, most of the current transformer-based architectures focus on 2D histopathological imaging. With the increasing application of transformers in histopathological image classification and prediction, we believe that more work will be put towards building 3D transformer models. Finally, the increasing popularity of hybrid transformers has recently gathered so much attention, as they have gained from both sides of transformers and conventional networks such as CNN and GNN.

#### Histopathological image segmentation

**Fig. 10:**
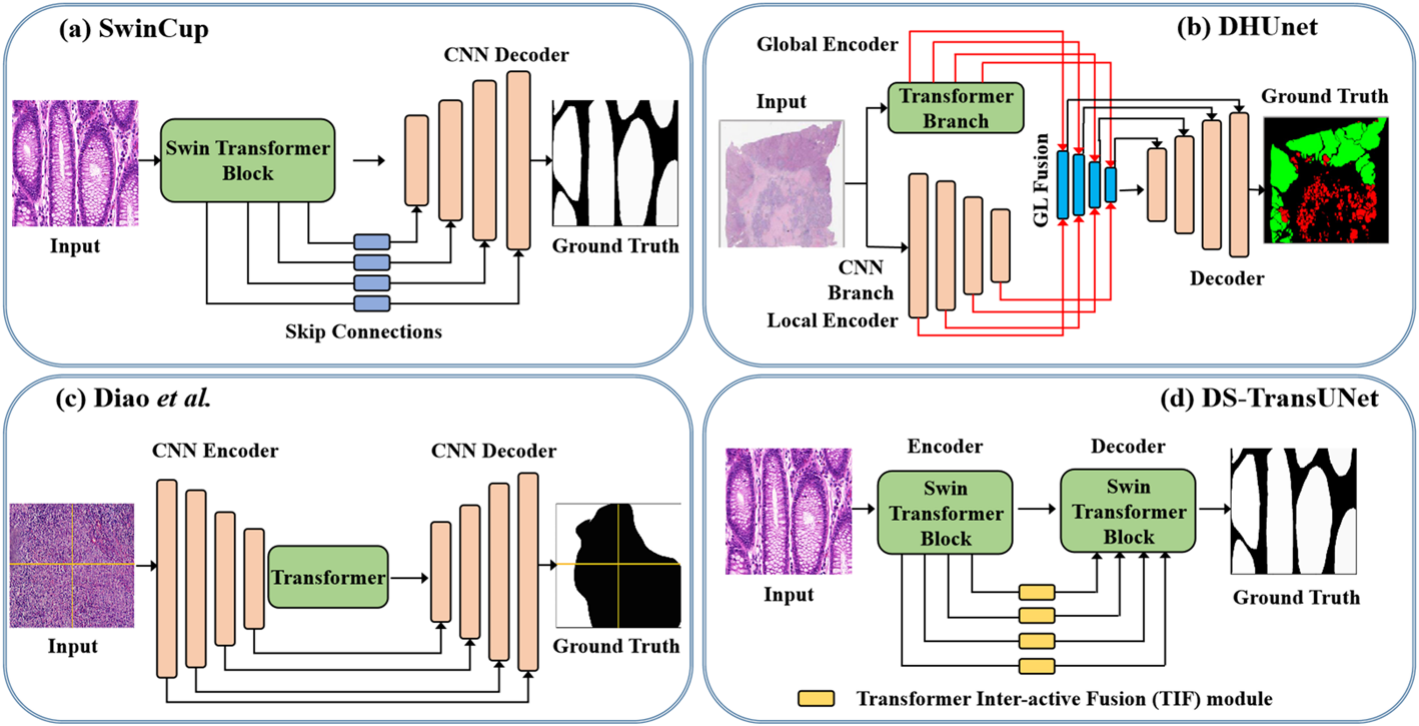
Some examples of SOTA transformer architectures for histopathological image segmentation

Semantic segmentation of tumor regions is a crucial task in histopathological image analysis. During segmentation, a region of a whole slide image (WSI) is used as input, and the model then segments the region using predetermined features. Despite recent developments in deep learning over the years, it was still a crucial and difficult task for researchers to segment the region of interest or cancerous region of histopathological images until the advent of vision transformers. Nowadays, transformer-based approaches have been used to solve a number of segmentation challenges, such as colon cancer segmentation [83], multi-organ nucleus segmentation [2], and nuclei segmentation [15, 50, 84, 85]. Some outstanding SOTA works are tabulated and detailed in Table 2, along with their associated network type, tissue type, dataset, challenge, highlight, etc. Figure 10 shows some examples of SOTA transformer architectures developed for histopathological image segmentation.

**Table 2:** Transformer applications in histopathological image segmentation

Method	Tissue	Dataset	Challenge	Highlight	DSC / IoU / F1 (%)
Swin-MIL [83]	Intestine	Custom	Image annotation and lack of related information between instances	Transformer-based weakly supervised approach	–/–/ 99.90
MCTrans [84]	Cell	Pannuke	Inability of CNN-based methods to model long-term dependencies	Multi-compound transformer with CNN	68.90/– /–
TSHVNet [85]	Cell	CoNSeP and Pannuke	Difficulties in differentiating various classes of nuclei and separating nuclear instances with high clustering,	Integration of multiattention modules (transformer and SimAM)	85.6 /– /82.00
Diao *et al.* [86]	Colon	NPC2020	Insufficient global context encoding	Transformer-based network using TransUNet	83.30/73.00 /–
DS-TransUNet [15]	Colon	GlaS	Ignoring the pixel-level intrinsic structural features inside each patch	Dual Swin transformer U-Net with standard U-shaped arch	87.19/78.45/–
TransAttUnet [87]	Colon	GlaS	Modeling long-range contextual dependencies and Computational costs	Transformer with Multi-level Attention-guided U-Net	89.11 / 81.13 /–
ATTransUNet [2]	Colon	GlaS and MoNuSeg	Heavy computational burden of paired attention modeling between redundant visual tokens	A transformer-enhanced hybrid architecture based on the adaptive token	89.63 / 82.55 /–
HiTrans [88]	Liver	PAIP 2019	The inherent heterogeneity of hepatocellular carcinoma	A hierarchical transformer encoder-based network	–/ / 75.13
TransWS [40]	Colon and breast	GlaS and Camelyon16	highlighting target regions roughly, sub-optimal solution and low efficiency	Transformer-based weakly supervised learning	– /–/ 85.20
TransNuSS [50]	Colon and breast	TNBC and MoNuSeg	The challenges of pre-training nuclei segmentation models with ImageNet due to morphological and textural differences	Self-supervised learning incorporated with vision transformer model	83.07 / 68.72 /–
NST [89]	Liver, Breast, Colon, etc.	GCNS and MoNuSAC 2020	The staining of WSI sections is not uniform and nuclei having different sizes and shapes	A gastrointestinal transformer-based network	79.60 / 66.30 /–
MedT [90]	Colon and cell	GlaS and MoNuSeg	Inherent inductive biases in CNNs and insufficiently annotated datasets	Gated axial-attention transformer-based model	–/ 69.61 / 81.02
SwinCup [48]	Colon and colorectal	GlaS	Inability of CNNs to model global context	Cascaded Swin transformer-based network	–/–/ 92.00
DHUnet [49]	Breast, liver, and lung	BCSS, WSSS4LUAD, etc.	Inability of the transformer model to capture fine-grained details in pathological images	Dual-branch hierarchical global–local fusion network	93.07 / 87.04 /–

**Fig. 11:**
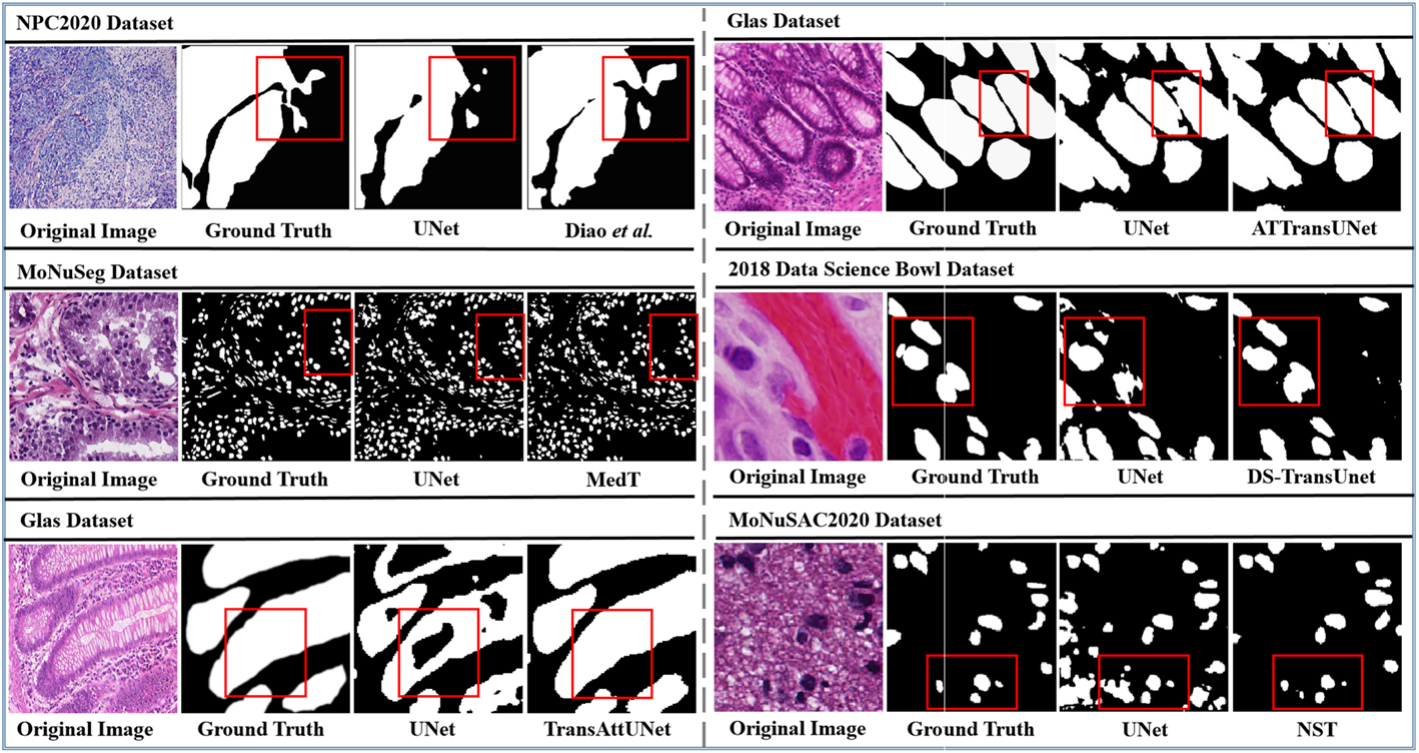
Examples of segmentation results of popular Unet architecture [38] and transformer-based models (ATTransUNet [2], DS-TransUNet [15], MedT [90], Diao et al. [86], TransAttUnet  [87], and NST [89]) on different histopathological datasets

The U-shaped CNN-based methods, often known as UNet [38], have obtained remarkable success in a number of histopathological image segmentation challenges. Besides, UNets are constrained in modeling long-term dependencies because of the convolutional layers present in them. Hence, in order to solve this challenge, researchers have made tremendous efforts over the years to develop high-performance hybrid transformers integrated with the UNet backbone. One of the most logical ways of inserting a transformer block into the U-shaped network is to place the entire transformer architecture between the encoder and decoder blocks so as to create long-range dependencies between high-level vision generalizations, as shown in Fig. 10. Some studies place the entire transformer architecture in the encoder part, while others place it in the decoder part. Methods such as TransNuSS [50], SwinCup [48], Diao et al. [86], DS-TransUNet [15], HiTrans [88], and DHUnet [49] [see Fig. 10(b)] are some examples of transformer-based U-shaped networks developed for histopathological image segmentation. In contrast to the various approaches mentioned above that incorporate transformer and U-shaped architectures within a single inference pathway, other studies looked into new ways of bridging transformers and CNNs for more accurate and robust segmentation. Although transformer-based architectures demonstrate the superiority of modeling long-range contextual information, their inability to capture local features still poses a lot of problems. Rather than cascading the transformer and convolution blocks, many studies recommend using the vision transformer and CNN as encoders that both accept histopathological images as input. After that, the embedded features are combined to link with the decoder. This approach benefits from simultaneously learning local and global information and then stacking representations sequentially [89]. Other than using U-shaped transformer-based architectures, some methods, such as MCTrans [84], TransAttUnet [87], MedT [90] and TSHVNet [85] applied multi-scaling techniques for histopathological image segmentation. In addition, pure transformer-based architectures can also be applied to a variety of histopathological image segmentation tasks. With the exception of the UNet network variations already mentioned, using the transformer in conjunction with convolution blocks, TransWS [40] introduced a transformer-based weakly supervised learning method without convolution layers. The proposed approach was basically used to address the issues of low efficiency and sub-optimal solutions as well as the challenge of producing a high-quality class activation map that identifies the precise and integral target, leading to insufficient activation and undefined boundaries. Qian et al. [83] built a weakly supervised approach that inserts the transformer architecture into the MIL module to encode long-term or global dependencies. Figure 11 shows some visual segmentation results obtained from various transformer networks against the popular Unet architecture on different histopathological image segmentation datasets.

In summary, from the research papers surveyed in this section, we can conclude that the histopathological image segmentation domain has been slightly impacted by transformer-based architectures since the inception of the first ViT architecture, as shown in Table 2. In comparison to other medical imaging fields, we strongly believe that this is due to a lack of annotated histopathological segmentation datasets and the high computational cost of training WSIs. As stated above, the high computational cost involved with mining features at multiple intensities obstructs the applicability of multi-scale networks in histopathological image segmentation tasks. These multi-scale networks make use of processing input image information at several levels and obtain significantly better performance than single-scale networks. As a result, building efficient transformer-based models for multi-scale processing needs better attention. Besides, most of the recently developed transformer-based architectures are pre-trained mainly on the ImageNet dataset for various downstream tasks. Hence, this technique is sub-optimal because of the huge domain gap between histopathological images and natural images. Recent ViT-based methods have largely focused on 2D histopathological image segmentation; therefore, building customized architectural frameworks by integrating temporal features for robust high-dimensional and high-resolution segmentation of WSI has not been fully investigated. Furthermore, with the development of ViT-based methods, we discovered that there is an urgent need to gather more varied and demanding histopathological image datasets. Although challenging and diverse datasets are also very important for evaluating the performance of transformers in other clinical settings, they are especially important for histopathological image segmentation because of the major influx of transformer-based approaches in this domain. To this end, we anticipate that these datasets will be crucial in determining the viability of ViT-based models for histopathological image segmentation.

#### Histopathological image detection and localization

**Fig. 12:**
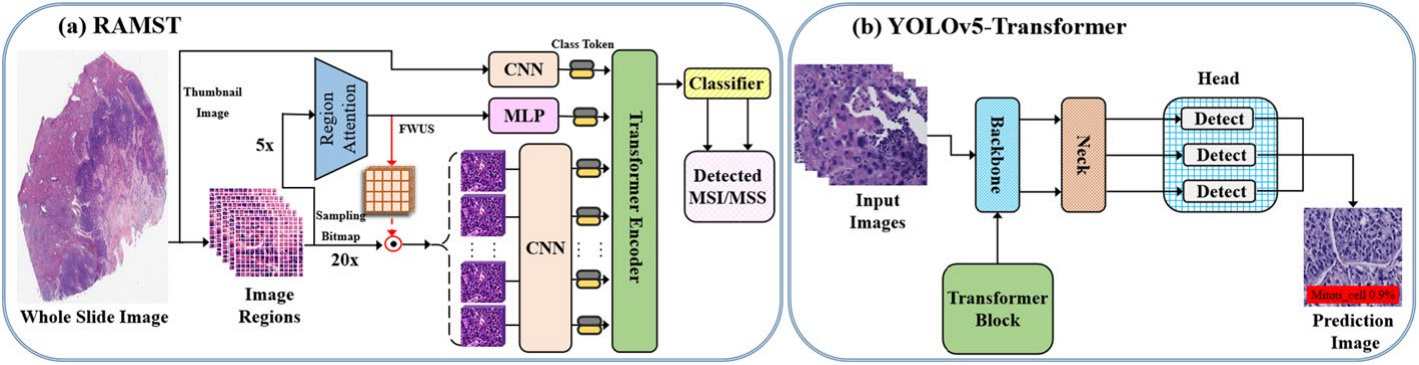
Some examples of SOTA transformer architectures for histopathological image detection

The word “detection” has different meanings across many domains. As we mentioned earlier, it is frequently referred to as disease identification or diagnosis in clinical domains, whereas in the technical field, it simply refers to determining whether lesions or diseases are present. However, disease detection in histopathological images is often referred to as a technique for locating instances of diseases in a specific image and identifying the potential region of a tumor, such as mitosis detection from breast cancer images, and is generally an important aspect of disease identification. Disease diagnosis is one of the most challenging tasks for clinicians, so it is important to have a reliable CAD technique that can serve as a second observer and potentially speed up the diagnosis process. Following the success of CNN-based methods in histopathological image detection and localization, there have been a few attempts recently to improve performance using transformer-based architectures. These techniques are primarily based on the detection transformer (DETR) [33]. Transformer architectures used for detection tasks involving histopathological images often incorporate CNN blocks, where CNNs are mainly used to mine features from images while the transformers are used to improve the mined features for other subsequent tasks. A few outstanding SOTA works are tabulated and detailed in Table 3. Figure 12 shows some examples of SOTA transformer architectures developed for histopathological image detection.

**Table 3:** Transformer applications in histopathological image detection and localization

Methods	Tissue	Dataset	Challenge	Highlight	ACC / F1 (%)
GasHis-transformer [19]	Stomach (Gastric)	HE-GHI-DS	Inability of CNN models to handle global information well	GasHis-transformer and LW-GasHis-transformer	97.97 / 97.97
PathTR [91]	Breast	CAMELYON16	Neglecting the intrinsic WSI global correlations among the patches	Context-Aware Memory ViT with a CNN Backbone	98.91 /–
PVTCB-Lymph-Det [92]	Colon, breast and prostate	LYSTO	Detecting lymphocytes automatically due to the presence of artifacts and morphological variations	Pyramid ViT-based network and convolution attention mechanism with ResNet-50	–/ 88.92
YOLOv5-transformer [93]	Breast, Colon, etc.	Custom	Accurate mitoses detection and morphological variations	Improved YOLOv5 transformer-based architecture	–/ 77.00
RAMST [94]	Stomach and colorectal	TCGA (CRC and STAD)	Unstable predictions caused by noisy patches and aggregation techniques	Joint regional attention and multi-scale transformer network	–
CB-HVTNet [95]	Colorectal, breast, etc.	LYSTO and NuClick	Insufficient feature representations	Channel-boosted hybrid ViT network	–/ 80.00
Hossain *et al.* [96]	Breast, etc.	TCGA and Custom	ViT-based network	ROI selection ViT-based network	96.10 /–
PersAM [18]	Lymph	Custom	Attention region estimation in digital pathological images	Personalized attention mechanism ViT network	83.13 /–

Recently, Chen et al. [19] proposed a multi-scale ViT-based approach that makes use of a position-encoded ViT framework and a CNN with convolutional operation to mine global and local information. To tackle the large-scale context overflow challenges, Wenkang et al. [91] developed a novel transformer-based technique that integrates global and local context within an end-to-end module. In addition, Ali et al. [92] introduced a transformed-based CAD system by making use of deep CNN networks based on channel boosting techniques. Takagi et al. [18] proposed a ViT-based personalized attention mechanism network for gigapixel WSIs with clinical records. Liaqat et al. [95] developed a channel-boosted hybrid ViT-based network that makes use of transfer learning techniques to build boosted channels and uses both ViT and CNN models to analyze cancerous images. As shown in Fig. 12, RAMST [94] makes use of joint region attention and a multi-scale transformer network to alleviate the unstable predictions caused by noisy patches and aggregation techniques in WSIs. YOLOv5-transformer [93] built an improved transformer architecture that integrates transformer into the YOLOv5 model for mitoses detection. Hossain et al. [96], on the other hand, built a region of interest (ROI) selection ViT-based architecture to speed up the analysis of histopathological images and improve the detection accuracy of cancerous regions. 

In summary, the number of new ViT-based architectures for the histopathological image detection and localization challenge task, as presented in Table 3 is lower than that of the classification task as reported in this survey paper. This is in comparison to the previous CNN-based methods that were promptly built for histopathological and other clinical detection tasks. Some recent medical research papers demonstrate that the generic, class-agnostic detection system of multi-modal ViT-based models pre-trained on other images rather than medical images performs horribly on histopathological and other clinical datasets. Hence, evaluating the performance of multi-modal ViT-based architectures by pre-training them on modality-specific histopathological WSI datasets is a good research direction to investigate in the future.

#### Histopathological image survival analysis and prediction

**Fig. 13:**
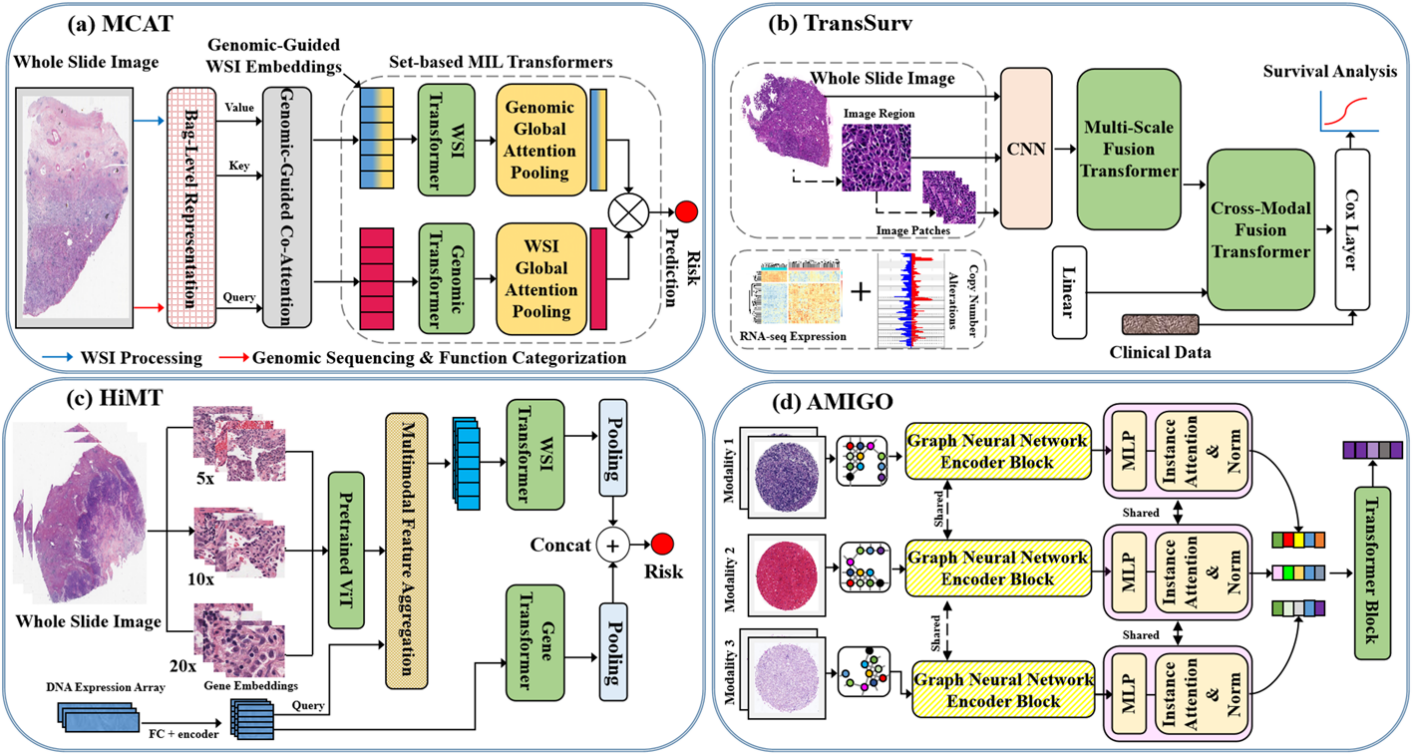
Some examples of SOTA transformer architectures for histopathological image survival analysis and prediction

Survival analysis and prediction is an arduous regression problem that aims to predict the time to an event, for example, the diagnosis of a disease or the relative risk of cancer death. Over the years, several techniques have been developed for survival analysis and prediction using histopathological WSIs. However, these techniques can be classified into two distinct classes: ROI-based and WSI-based approaches, respectively. Due to the high cost of computational resources, the majority of the existing literature has concentrated on regions of interest (tiles) chosen by pathologists from WSIs. Nowadays, a number of methods for histopathological image analysis have been proposed for a wide range of downstream tasks, using the detailed and dense annotations on WSIs. Recently, transformer-based architectures have demonstrated outstanding performance in predicting survival rates. A few outstanding SOTA works are summarized and detailed in Table 4. Fig. 13 shows some examples of SOTA transformer architectures developed for histopathological image survival analysis and prediction.

**Table 4:** Transformer applications in histopathological image survival analysis and prediction

Method	Tissue	Dataset	Challenge	Highlight	C-index (%)
TransSurv [97]	Colorectal	TCGA-CRC and NCT-CRC-HE	Inability of the previous models to extract useful predictive features from the multi-modal data	Transformer-based multi-modal feature fusion network	82.20
PG-TFNet [98]	Colorectal	TCGA-CRC	Inability to make use of the powerful representation learning capabilities of the neural networks	Transformer-based multi-modal feature fusion network	81.60
ESAT [99]	Lung	NLST and CHCAMS	Using a pre-selected subset of main patches or patch clusters as input instead of using the entire WSIs	Make use of the ViT backbone with convolution operations.	73.00
MCAT [26]	Bladder, Breast, Lung, Uterine	BLCA, UCEC, BRCA, BMLGG, LUAD	Computational complexity and large data heterogeneity gap between genomics and WSIs	Multimodal Co-Attention Transformer for Survival Prediction	65.30
HiMT [100]	Bladder, Breast, Lung, Brain, etc.	BLCA, BRCA, UCEC, LUAD, LGG, etc.	High computational cost of extracting patches from WSIs, which results in a large bag size	Hierarchical-based multi-modal Transformer framework	67.30
MaskHIT [82]	Breast, Lung, etc.	TCGA	Huge number of network parameters and insufficient labeled data	Masked pre-training of Transformers	61.20
SURVPATH [101]	Breast, Bladder, Stomach, etc.	TCGA	Capturing dense multimodal interactions between different modalities	Memory-efficient multimodal Transformer	62.90
Surformer [102]	Bladder, Breast, Lung, etc.	TCGA (BLCA, BRCA, LUAD, etc.)	Weak interpretability problems of the previous computational pathology model	Pattern-perceptive survival Transformer-based Network	68.70
HVTSurv [103]	Bladder, Breast, Lung, etc.	TCGA (BLCA, BRCA, LUAD, etc.)	The challenges of exploring contextual, spatial, and hierarchical interaction in the patient-level bag	Hierarchical ViT-based architecture	63.40
HMCAT [104]	Low Grade Glioma	TCGA-GBMLGG	The significant disparity between the spatial scales of radiology images and WSIs	Hierarchical multimodal co-attention transformer-based network	79.60
AMIGO [3]	Ovarian and bladder	InUIT and MIBC	ignoring specific details regarding the individual cells in a tile image	Sparse multi-modal graph Transformer-based network	61.00
SeTranSurv [25]	Breast, Lung, Ovarian	OV, LUSC, and BRCA	Ignoring the important role of spatial information in patches and the correlation between patches and WSIs	Integration of patch features through self-supervised learning and Transformer	70.50

Transformer-based methods such as HiMT [100], MCAT [26], PG-TFNet [98], TransSurV [97], and SURVPATH [101] combine genomic data and histopathological images for survival analysis and prediction. As shown in Fig. 13, MCAT [26] introduced a multimodal co-attention Transformer network to learn an interpretable, dense co-attention mapping among genomic features and WSIs constructed in an embedding space. TransSurV [97] makes use of a Transformer-based multi-modal feature fusion network to extract useful predictive features from the multi-modal data. HiMT [100] introduced a hierarchical transformer-based network to mine the instant-level tile features at random from WSIs with varying magnification levels. AMIGO [3] created a multi-modal graph transformer architecture that predicts patient survival based on multi-modal histopathological images and shared related data. In addition, Huang et al. [25] designed a transformer technique for survival prediction based on the combination of tile features via an self-supervised learning (SSL) approach and a transformer. Shen et al. [99] make use of an explainable survival analysis framework coupled with a convolution-involved ViT-based network. More recently, Jaume et al. [101] introduced a memory-efficient multimodal-based transformer architecture that combines patch tokens and transcriptomics for patient survival prediction. Wang et al. [102] developed a pattern-perceptive survival transformer-based network that can statistically interpret the predictions as well as directly quantify the important histopathological patterns. HMCAT [104] introduced a hierarchical multi-modal co-attention Transformer-based network that addresses the challenges of the large size of histopathological WSIs and the significant disparity between the spatial scales of radiology images and histopathological WSIs. Shao et al. [103] make use of a hierarchical ViT-based architecture to completely investigate the contextual, spatial, and hierarchical relationships in the patient-level bag. 

In summary, the number of new ViT-based architectures for the histopathological image survival and prediction task is lower compared to that of the classification tasks reported in this paper. This is in comparison to the previous CNN-based methods that were promptly built for histopathological and other clinical survival and prediction tasks. It is also important to note that despite the fact that there are several survey papers covering the applications of CNNs in histopathological image analysis [4–6], none of these studies have recently covered the use of Transformer architectures in survival analysis and prediction, despite the outstanding performance that these architectures have demonstrated over the last few years. We anticipate that this part will be a useful tool for researchers in this domain. In addition, we will briefly discuss some problems with transformer-based architectures for survival analysis and prediction below, along with some interesting future prospects. 

As shown in Table. 4, transformer-based survival analysis and prediction architectures mostly rely on the concordance index metric (i.e., c-index) to evaluate the performance of the networks, which sometimes fails to accurately reflect clinical efficacy. Since the researchers currently depend only on the c-index metric as an evaluation metric, we believe that further effort is needed to develop more accurate clinical evaluation indicator to speed up the adoption of transformer-based survival analysis and prediction in clinical domains. Again, some of the transformer-based architectures surveyed in this paper make use of histopathological images and genomic records for survival analysis and prediction. Hence, generating reports from other clinical or medical domains has its own challenges due to their unique nature and varied features. Besides, a few histopathological datasets, like TCGA ,^1^ are available that consist of different cancer types together with clinical records. This dataset has the potential to be a valuable baseline for evaluating the performance of future multimodal transformer-based architectures for survival analysis and prediction. We suggest that in the future, transformer-based architectures tailored to particular tissues to predict patient survival should be investigated, with a focus on building challenging and varied datasets of different tissues.

#### Histopathological image representation

**Fig. 14:**
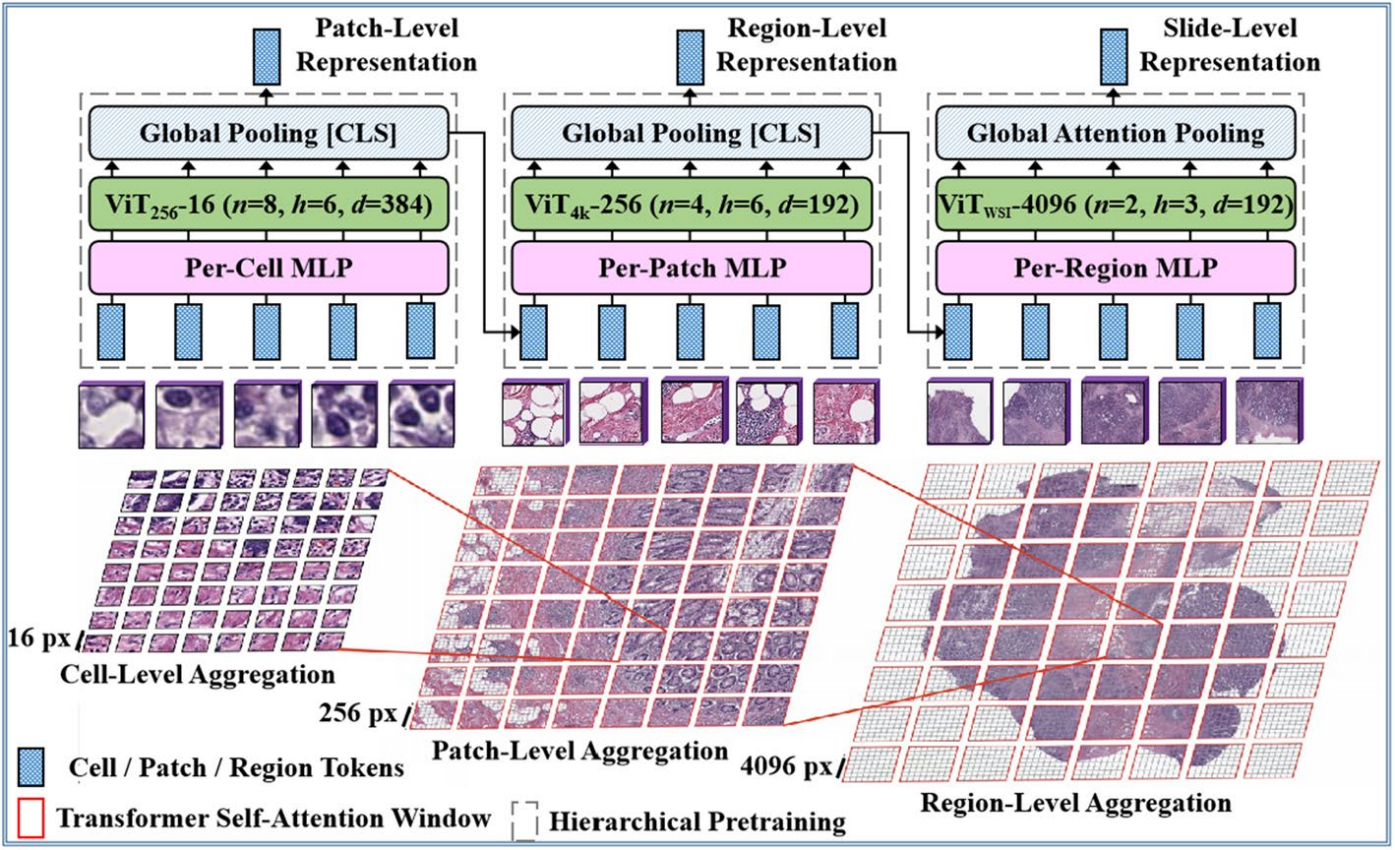
A schematic illustration of the HIPT [22] transformer architecture for histopathological image representation

Due to memory and processing time constraints, histopathological images are often divided into smaller tiles (such as 256 $\times$ 256 pixels), and features are then mined concurrently from each tile. The representation of a histopathological WSI using information from multiple tiles, however, is a developing field of study with limited results that have been published, particularly in the context of clinical prediction and prognosis. Recently, several studies have been developed for learning multi-scale representations of images using transformer-based models, which can also be employed in convolutional pipelines in order to construct global representations of images. A few outstanding SOTA works are tabulated and summarized in Table 5. Figure 14 shows an example of the SOTA transformer architecture developed for histopathological image representation.

**Table 5:** Transformer applications in histopathological image representation

Method	Tissue	Dataset	Challenge	Highlight	ACC / AUC (%)
CD-Net [105]	Breast, Lung	TCGA (LUAD and LUSC)	Inability to leverage the rich multi-resolution information	Transformer-based pyramidal context-detail network	91.10 / 95.80
DSCA [106]	Lung, breast and brain	NLST, TCGA (BRCA and LGG)	High computational complexity and unnoticed semantic gap in multi-resolution feature fusion	A dual-stream Transformer network with cross-attention framework	-
HIPT [22]	Breast, lung, stomach, cell	IDC, LUAD, CCRCC, PRCC, CHRCC, and STAD	The structure of phenotypes in tumors and learning a good representation of a WSI	Hierarchical image pyramid Transformer with two levels of self-supervised learning	–/ 98.00
HEAT [107]	Colon, breast and esophageal	CAMELYON16, TCGA (COAD, BRCA, and ESCA)	The challenges of extracting diverse interactions between various cell types	Heterogeneous-graph edge attribute Transformer-based network	99.90 / 99.90
H2T [21]	Lung, breast and kidney	TCGA-NSCLC, CPTAC-LUAD, etc., BRCA, RCC and ACDC	High discordance on how a tissue sample and higher predictive power that comes at the cost of interpretability	Handcrafted histological Transformer-based network for unsupervised representation WSIs	-
ViT-AMCNet [20]	Laryngeal, breast, brain	Laryngeal cancer, breast cancer, brain cancer	Problems of poor transformer generalization bias and poor AMC interpretive ability	ViT-based network with adaptive model fusion and multi-objective optimization	95.14 / 96.17

In order to learn high-resolution image representations from histopathological images, HIPT [22], made use of a ViT-based hierarchical image pyramid network, CD-Net [105] proposed a Transformer-based pyramidal context-detail network, and H2T [108] employed a handcrafted histological Transformer. As presented in Fig. 14, HIPT [22] uses two levels of self-supervised learning to take advantage of the natural hierarchical structure present in histopathological WSIs. The proposed architecture was pre-trained across 33 different cancer types by making use of 10,678 histopathological slides, 104 M 256 $\times$ 256 images, and 408,218 4096 $\times$ 4096 images. In addition, DSCA [106] built a dual-stream Transformer architecture with cross-attention to address the challenges of the unseen semantical disparity in multi-resolution feature fusion and the high computational complexity of histopathological WSI visual representation. ViT-AMCNet [20] makes use of an end-to-end transformer-based network with adaptive model fusion and a multi-objective optimization technique to address the challenges of poor interpretability and weak inductive bias ability for the laryngeal tumor grading task. Chan et al. [107] built a heterogeneous-graph edge attribute transformer-based network that can benefit from both node and edge heterogeneity. 

In summary, since the number of publications and transformer applications in histopathology image representation is currently limited, as shown in Table. 5, it is challenging to draw any conclusions at this time. However, as the current transformer-based architectures give better results on histopathological image representation tasks, we anticipate further development in this domain in the near future.

#### Transformer applications in other histopathological imaging tasks

This section briefly discusses the use of transformers in other histopathological gigapixel image domains, such as cross-modal retrieval analysis, image generation, image synthesis, and so on. Dingyi et al. [23] introduced a cross-modal retrieval dual-transformer architecture that can simultaneously execute four retrieval tasks at a time for the histopathology dataset across diagnosis reports and WSIs, respectively. MedViTGAN [24] developed a conditional GAN transformer-based network that can aid researchers in producing synthetic histopathological images for other downstream tasks in an end-to-end approach. In addition, a ViT-based network to enhance the use of contextual information found in histopathological images was proposed in  [109]. The network is made up of two variations of ViT-based architecture (PREViT and ClusterViT) to improve the local context of the tissue patch features by adding prior knowledge to the network. Xu et al. [110] developed a Transformer architecture for high-quality histopathological image synthesis that combines ViT and diffusion autoencoders. The authors introduced a conditional denoising diffusion implicit model (DDIM) into the architecture, which was improved by integrating a ViT model as a semantic encoder, allowing it to comprehensively encode sophisticated phenotypic layouts particular to histopathology. 

To this end, since the current transformer-based architectures give better results on other histopathological image tasks, we anticipate further development in these fields in the near future.

## Discussion

Transformer architectures have been effectively used in a wide range of clinical tasks, including histopathological image analysis, as demonstrated in Tables 1, 2, 3, 4, and 5. Despite their strong performance due to the attention mechanisms incorporated into them, there are a number of challenges that could prevent transformer models from performing well, especially in real-world clinical applications. One of these challenges is the lack of supervised clinical information provided by experts in order to develop a supervised Transformer architecture, which is critical in training a transformer model. Therefore, in this section, we will discuss the recent research directions in addressing this challenge using the SOTA transformer models in different learning settings and also compare transformers with CNNs based on recently published papers.

### Different learning settings with transformer architectures

In this section, we present and discuss different learning settings that are often used with transformer architectures for histopathological image analysis, including weakly supervised learning, self-supervised learning (SSL), multi-task learning (MTL), and multi-modal learning (MML). 

(i) Weakly supervised learning for histopathological imaging: The creation of a weakly supervised transformer-based architecture addresses the urgent need for histopathological image annotation, which is generally labor-intensive and also time-consuming. On the other hand, weakly supervised learning is an arduous task where a huge number of instances occur within each bag while only a slide label is provided. Multiple instance learning (MIL), which is also a subset of weakly supervised learning, has shown better results in a number of downstream tasks in previous studies. Despite recent improvements, they still have some drawbacks. One is that they concentrate on the regions that are easily distinguished as positive for the diagnosis while neglecting the positives that make up a small proportion of the WSI. For the purpose of obtaining more discriminative features, several studies have developed a number of weakly supervised approaches for histopathological image analysis, including segmentation [40, 83], and classification [2, 17, 45, 56, 111] tasks. Besides, the weakly supervised ViT-based MIL technique was further adopted for colorectal cancer lymph node metastasis (LNM) prediction [56] and can be used to reduce the doctor’s workload and accelerate diagnostic operations. 

(ii) Self-supervised learning for histopathological imaging: Supervised learning techniques heavily rely on pathologists to manually annotate several regions on WSIs before they can be used to train any network. However, this approach often requires a significant amount of annotated datasets for transformer architectures to be successfully trained in many computer vision tasks, and some of these datasets are uncommon in real clinical settings. Therefore, such problems were addressed by self-supervised learning (SSL) techniques. The primary objective of SSL is to enhance the performance of different downstream tasks by conveying knowledge from the associated unsupervised upstream task and pre-training the network by making use of its self-contained features in the untagged data. The standard method for training SSL ViT architectures involves pre-training the architecture primarily on ImageNet and then fine-tuning it on the targeted histopathological image dataset. This will generally improve the performance of transformers in contrast to CNNs and allow for the attainment of SOTA accuracy. A significant amount of studies have attempted to apply the SSL approach for a variety of objectives in histopathological image analysis, including representation [22], classification [64, 75], and survival analysis and prediction [25] tasks. 

(iii) Multi-task learning for histopathological imaging: Multi-task learning (MTL) is a technique in which a shared network learns multiple tasks at the same time. It has shown better performance than single-task learning techniques in increasing the learning capabilities of a deep learning architecture. Such techniques offer many benefits, such as preventing overfitting through the use of shared representations, speeding up learning by utilizing auxiliary information, and increasing data efficiency. However, building transformer architectures with multiple tasks assists in increasing the network generalization ability, which is crucial in histopathological image analysis. Recently, MTL has been commonly applied to transformer-based networks to tackle various downstream tasks in computer vision, and a commonly used technique is to combine a segmentation and classification task into a single network [40, 85]. In addition, Ali et al. [92] introduced a transformed-based CAD system by making use of deep CNN networks based on channel boosting techniques to improve the learning capability of the entire network. Wang et al. [45] built a weakly supervised transformer architecture by integrating graph neural networks and transformers for basal cell carcinoma classification and detection. 

(iv) Multi-modal learning for histopathological imaging: Multi-modal learning (MML) is an approach that aims to build and develop models that can integrate data from multiple modalities, such as image data, genomic data, and clinical records. Over the years, research advancements in MML have grown rapidly in a number of computer vision tasks, particularly histopathological image analysis. It involves utilizing a single model to learn representations from various modalities. Using data from multiple modality sources, on the other hand, provides additional clues for disease diagnosis. Several studies have investigated the integration of genomic data and histopathological images for survival analysis and prediction using transformer-based architectures [26, 100, 101]. Takagi et al. [18] proposed a ViT-based personalized attention mechanism network for histopathological images with clinical records. AMIGO [3] created a multi-modal graph transformer architecture that predicts patient survival based on multi-modal histopathological images and shared related data. Cai et al. [60] created a frequency-domain transformer architecture that integrates frequency and spatial domains for histopathological lung cancer image analysis and subtype determination. 

In summary, transformer architecture is regarded as a promising technique for fusing computer vision and NLP tasks. However, there is still a need to develop more accurate and robust CAD systems for real-time clinical settings where multiple data types, such as imaging, clinical, and laboratory records, are regarded as multiple sources of information.

### Comparison of transformers and CNNs on different downstream tasks

Over the years, CNN-based architectures have been dominant in many research fields prior to the development of vision transformers (ViTs), including the field of histopathological image analysis. Many studies have also been conducted in this domain to ascertain whether CNN-based architectures can still work on ViT-based architectures. Recently, ViT-based architectures have been shown to be capable of producing better results than CNNs, especially when pre-trained on a large number of datasets. In comparison to CNNs, ViTs have a weaker inductive bias, and as a result, they allow for more flexible feature detection. The performance comparison between ViTs and CNN-based models has received tremendous attention, as ViTs have excelled in a number of benchmarks, as shown in Fig. 11. Nguyen et al. [112] comprehensively evaluated six frequently used Transformer-based architectures for cancer segmentation. The results obtained, with the exception of Swin-UNet [113], show that Transformer-based architectures typically outperform CNN-based techniques because of their capacity to encode global context. Besides, this is one of the first studies to systematically compute the performance of transformer-based approaches on histopathological image segmentation. For the task of tumor detection and tissue type identification in digital pathology WSIs, Deininger et al. [114] compared the patch-wise classification result of the ViT DeiT-Tiny to the SOTA CNN-based ResNet18 model. Due to the limited number of annotated slide images, the authors further compared the two architectures by pre-training them on a large number of unlabeled WSIs using SOTA self-supervised techniques. The obtained results demonstrate that the ViT slightly outperformed the ResNet18 for three out of the four tissue types investigated in the study for tumor detection, while the ResNet18 architecture slightly outperformed the ViT for the remaining tasks. In addition, Springenberg et al. [115] conducted an extensive evaluation of deep learning architectures for histopathological image classification by comparing Transformers and CNNs, respectively. The study produced concrete architecture recommendations for medical practitioners as well as a generic approach for quantifying architecture quality based on complementary conditions that can be applied to future network architectures. 

In summary, many previous studies and SOTA on histopathological image analysis have not completely shown that transformer-based architectures can outperform CNN-based architectures in all ramifications, especially in few-shot and low-resolution histopathological image analysis. Thus, developing hybrid architectures with convolutions, similar to approaches in computer vision, has been adopted in most current research works. In addition, apart from the excellent results achieved in most publications surveyed in this paper, as demonstrated in Tables 1, 2, 3, 4, and 5, transformer architectures are computationally expensive and require a large amount of data for training. Therefore, we anticipate further development in reducing transformer computational complexity in the near future.

### Other challenges and future directions

We primarily reviewed the current SOTA Transformer-based methods for histopathological image analysis. There are still a number of open challenges to be addressed in the future, despite the excellent and outstanding results produced. (1) The first challenge is the intensiveness of annotations. Transformer-based architectures often need a large number of annotated datasets and can produce better results when trained on huge datasets, but their performance reduces when data or annotations are limited. To solve this problem, SSL techniques offer better and more interesting solutions. Transformers, on the other hand, can improve their capacity for representational learning by making use of unlabeled data and proxy tasks like reconstruction and contrastive learning. A significant number of studies have applied the self-supervised approach for a variety of objectives in histopathological image analysis [22, 25, 64, 75] and have shown better performance. Some of these approaches have demonstrated that training networks using large-scale unlabeled 2D images is advantageous when fine-tuning them with small-scale datasets. However, we find that pre-training is computationally expensive, and future research should focus on simplifying and analyzing the efficiency of the pre-training model as well as fine-tuning it for small-scale datasets. (2) The second challenge is the scalability of the task. The heterogeneous nature of histopathological images makes representational learning very difficult. Studies in the past have mainly concentrated on resolving specific histological tasks, and transformer architectures perform better at learning heterogeneous tasks, especially when SSL techniques are adopted [22]. Again, the advanced scaling operations also give the transformer-based architectures the capacity to handle multi-domain and multi-scale tasks [22]. In addition, networks may fit a variety of datasets by scaling up transformer architectures, and researchers can modify a network at training time to move from a low-data scheme to larger dimensions. (3) The third challenge is the scalability of the data. Most ViT-based architectures, such as the original ViT [8], perform poorly when trained on small-scale datasets because they lack inductive bias. However, If there is enough training data, transformer architectures can overcome inductive bias challenges by employing different pre-training techniques. Besides, pre-training techniques [22, 69, 82] have also shown better performance in increasing the generalization ability of transformer architectures for histopathological imaging. Moreover, gathering large-scale datasets in the histopathological imaging domain is sometimes impractical due to time-consuming manual annotations and patient privacy concerns. Since gathering large-scale datasets across different imaging modalities still poses a lot of challenges, it is therefore essential to build transformer architectures that are less data-demanding for histopathological imaging applications by incorporating inductive bias mechanisms into transformer models, and we hope to see further research addressing this challenge in the near future. 

(4) The fourth challenge is computational complexity. As shown in the previous sections, transformer-based architectures are computationally expensive due to the computation of the self-attention mechanism, which is usually quadratic to the size of the input image. This issue appears to be less of a problem with natural images, but with histopathological images, it is a significant difficulty. Again, this is because histopathological images such as WSIs come in gigapixels and are larger in size compared to natural image datasets. Unlike natural images such as ImageNet, which have fewer pixels, histopathological WSIs can be as huge as 150,000 x 150,000 pixels [22]. Compared with training strategies used for natural imaging models, transformer-based architectures for histopathological image analysis are typically more compacted and sometimes trained using patched input or even smaller batch sizes. The majority of SOTA transformer-based methods for histopathological image analysis are either built upon the already-existing transformer networks [15, 48, 51, 75] or make use of CNNs for feature extraction before being fed into a transformer [56, 61, 63, 81, 82]. Moreover, several studies have suggested that Softmax may be circumvented to linearize the computation of the self-attention mechanism, although none of these techniques have been used and applied to histopathological imaging yet. Therefore, we hope to see further research in this particular direction. (5) The fifth challenge is the combination of data from different sources. An emerging research area such as imaging genomics has opened up new possibilities for cancer detection and prediction. Using data from multiple modality sources, on the other hand, provides additional clues for disease diagnosis. Some of the Transformer-based architectures surveyed in this paper make use of histopathological images and genomic records for different downstream tasks [100, 101]. However, generating reports from other clinical or medical domains has its own challenges due to their unique nature and varied features. Therefore, how to properly incorporate data from multiple sources for more accurate disease identification and prediction is another interesting and promising future research direction. (6) The sixth challenge is the black box and its interpretability. Over the years, several studies have been conducted on histopathological imaging using various deep learning techniques. Deep learning methods such as CNN sometimes function as black box solutions and are typically more difficult to explain. transformers, on the other hand, make use of a self-attention mechanism that imitates some human behaviors but still functions as a black box and is unable to reveal how different factors are combined to generate results. Given the importance of network interpretability in histopathological image analysis, it is critical to investigate the interpretability of transformer-based architectures. One of the common methods for visualizing Transformer architectures is to calculate relevancy scores from either single or shared attention blocks. The MHSA module in transformer architectures establishes a direct link between tokens, providing an intuitive guide for decision-making. Recently, visual language pre-training [69] has also been adopted for histopathological imaging, and the majority of the WSI-level diagnosis or prediction networks are computed in a black box, making it impossible for humans to understand which region of the slide has the greatest influence on the final prediction. Hence, in order to make the networks more understandable, it is preferable to construct a transformer-based architecture that can identify discriminant patches from the histopathological WSI that generate clinical or medical results.

## Conclusion

Transformer architectures are now dominating almost all of the field of computer vision, with a rapid increase in the field of histopathological imaging. In this survey paper, we carry out a thorough review of the applications of transformer architectures in histopathological image analysis. In particular, we survey the applications of transformers in histopathological image classification, segmentation, detection, survival analysis and prediction, and representation and discuss their drawbacks. We found out that the majority of the existing transformer architectures can be naturally and easily applied to histopathological imaging challenges without significant modifications. As a matter of fact, many advanced approaches such as multi-task learning (MTL), weakly supervised learning, multi-modal learning (MML), and model enhancement across various domains are rarely investigated. In addition, we also provided unsolved research problems for further investigation. To this end, despite the outstanding performance of the transformer-based architectures in a number of papers reviewed in this survey, we anticipate that there will be much more exploration of transformers in histopathological image analysis to further increase the efficiency of clinicians, decrease subjectivity, and enhance patient safety. Moreover, the majority of the diseases reviewed in this paper focused more on histopathological image analysis, and it is expected that in the future, it will be extended to other imaging modalities where multiple data types, such as imaging, clinical, and laboratory records, are regarded as multiple sources of information. We hope that this survey paper provides readers in this domain with a comprehensive idea of transformers.

## Data Availability

Not applicable.

## Footnotes

1 https://www.cancer.gov/tcga.
